# Generative Landscapes and Dynamics to Design Functional Multidomain Artificial Transmembrane Transporters

**DOI:** 10.1021/acscentsci.5c00708

**Published:** 2025-07-10

**Authors:** Fernando Montalvillo Ortega, Fariha Hossain, Vladimir V. Volobouev, Gabriele Meloni, Hedieh Torabifard, Faruck Morcos

**Affiliations:** † Department of Chemistry and Biochemistry, 12335 University of Texas at Dallas, Richardson, 75080 Texas, United States; ‡ Department of Biological Sciences, 12335 University of Texas at Dallas, Richardson, 75080 Texas, United States; ¶ Departments of Bioengineering and Physics, 12335 University of Texas at Dallas, Richardson, 75080 Texas, United States; § Center for Systems Biology, 12335 University of Texas at Dallas, Richardson, Texas 75080, United States

## Abstract

Design and synthesis of functionally active artificial proteins is challenging, as it requires simultaneous consideration of interconnected factors, such as fold, dynamics, and function. These evolutionary constraints are encoded in protein sequences and can be learned through the latent generative landscape (LGL) framework to predict functional sequences by leveraging evolutionary patterns, enabling exploration of uncharted sequence space. By simulating designed proteins through molecular dynamics (MD), we gain deeper insights into the interdependencies governing structure and dynamics. We present a synergized workflow combining LGL with MD and biochemical characterization, allowing us to explore the sequence space effectively. This approach has been applied to design and characterize two artificial multidomain ATP-driven transmembrane copper transporters, with native-like functionality. This integrative approach proved effective in revealing the intricate relationships between sequence, structure, and function.

## Graphical abstract

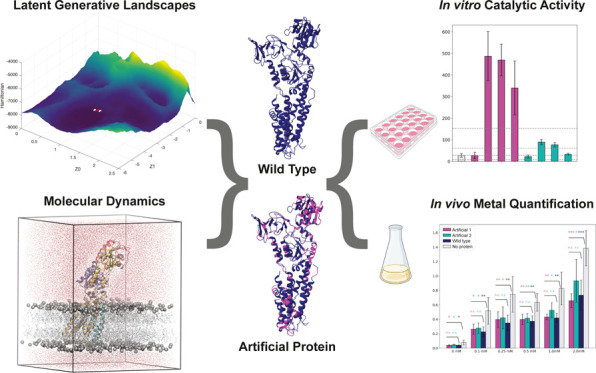

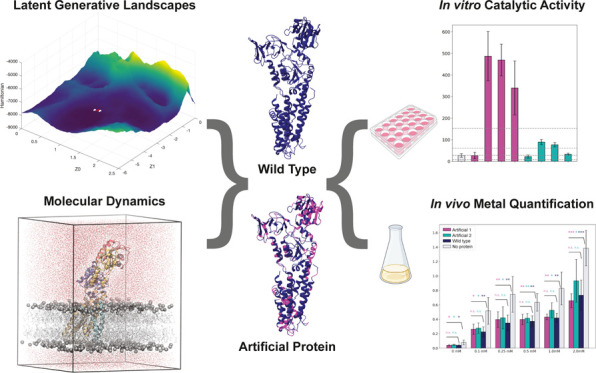

## Introduction

Protein design is becoming a cornerstone of modern biotechnology, with possible applications spanning from therapeutic drug design to industrial biocatalysis. Despite its potential, the immense combinatorial space of possible sequences and the challenge of preserving crucial interactions for functional and structural stability make the design process time-consuming and computationally challenging. However, recent advances in machine learning (ML) along with increasing sequence data availability have revolutionized our ability to model biological systems from evolutionary clues. Current applications range from protein structural prediction to the design of novel proteins with enhanced functionality (such as modifying antibody binding affinity to their respective targets or improving catalytic capacity of enzymes) and stability.^1–10^

Researchers have been exploring diverse ML strategies in protein design, ranging from sequence-based and sequence-labeling models to structure-based and hybrid approaches.^11,12^ Among these techniques, latent generative models, such as Variational Autoencoders (VAEs), have been only recently explored in the context of biological systems.^3–5,13^ We introduce a framework that learns from extant protein sequence data and harnesses the reconstructive ability of VAEs in conjunction with Direct Coupling Analysis (DCA) to produce maps of generated sequences known as latent generative landscapes (LGLs).^14^ These landscapes model protein sequence-function relationships, capturing sequence diversity within a family while assessing functional integrity, offering a powerful tool for protein design.

When designing proteins, it is important to preserve crucial motifs essential for protein function, as well as highly coupled interacting residues that form interdependent networks critical for the protein’s behavior, even beyond conserved sites. A key strength of the LGL framework lies in its ability to learn these key interactions from only protein-family sequences, scoring generated sequence variants more favorably (greater negative value) when such interactions are preserved.^14^ However, relying solely on sequence-based approaches can limit our understanding of intricate interdependencies within proteins, particularly in complex membrane-embedded nanomachines in which catalysis and multidomain coupling are central to protein function. Previous studies have successfully integrated molecular dynamics (MD) simulations with fitness landscape-based design to create functional soluble proteins.^15,16^ A less explored area is its application to integral membrane proteins. Among some successful examples are the work of DeGrado^17^ and Barth,^18^ which required the integration of diverse computational strategies, underscoring the difficulty of designing such systems. For this reason, coupling the LGL method with MD simulations allows for a deeper exploration of how interacting residues and novel mutations influence the protein’s conformational dynamics, domain crosstalk, and functional behavior. This integrated computational approach can bridge the gap between prediction and experimental success. Rather than conducting experiments on numerous designed sequences to identify functional ones, it enables us to achieve remarkable success with a carefully selected subset. This, in turn, allows for dedicating more resources to in-depth validation and characterization of the most promising candidates, an aspect often lacking in currently available protein design studies.

The P-type ATPase superfamily of transmembrane transporters is crucial for cellular homeostasis, coupling ATP hydrolysis to substrate transport across membranes against their electrochemical gradient.^19,20^ These large, multidomain transmembrane proteins rely on intricate crosstalk between multiple cytosolic and transmembrane domains. Their mechanism couples ATP hydrolysis with auto­(de)­phosphorylation to trigger dramatic and sequential conformational rearrangements in the cytosolic domains. This results in transmembrane helices movements, which in turn facilitate substrate translocation across the membrane lipid bilayer.^21,22^ Within this superfamily, the P_1B_-type ATPase subfamily plays an essential role in maintaining transition metal homeostasis, where the P_1B‑1_-type ATPases stand out as the ubiquitous Cu­(I) exporters in all organisms.^23^ Thus, the complexity, functional diversity, and relevance of this system make it an ideal candidate to test the robustness of the synergized protein design strategy.

Here, we apply an interdisciplinary approach to efficiently design artificial P_1B‑1_-type ATPase transporters that maintain their quintessential coupled multidomain complexity and multifunctional properties. First, we employ the LGL framework to produce novel variants of the transporter family, decoding few from the vast sequence space based on favorable Hamiltonian metric and the presence of key subgroup-specific motifs essential for function. Next, we leverage MD to assess the structural stability and dynamics adherence to the expected catalytic scheme. For these generated variants, we demonstrate coupled domain movements crucial for catalytic function, comparable to those identified in the wild-type (WT) protein(s). Finally, we experimentally characterize the preservation of multifunctional properties encompassing membrane embedding, proper fold, copper translocation, and *in vivo* cellular protection from copper toxicity. Our design of complex transmembrane transporters that integrate seamlessly into a lipid bilayer and maintain full functional fidelity advances previous attempts in this field.^24–27^ The rate of success on the selected designs is notable, something rare for sequences with hundreds of mutations, opening new possibilities for creating tailored proteins with specific functional characteristics through an efficient, sequence-driven approach synergized with molecular dynamics.

## Results and Discussion

### Latent Generative Landscape Produces Nonextant Transporter Sequences

The LGL framework enables visual and quantitative exploration of the sequence energy landscape, enhancing mutational analysis, the study of phylogenetic relationships, and identification of different functional clusters.^14^ However, the generative ability to produce complex new protein sequences with desired features has been less explored. The incorporation of the Hamiltonian metric, determined from key interactions learned within the protein subfamily using DCA (see Methods), hints at relative functional fitness. This definition of fitness incorporates various aspects of this type of multifunctional transmembrane proteins, such as selective transport of specific substrates, proper energy transduction, coupled conformational changes necessary for activity modulation, and intercommunication between domains. These nanomachines couple ATP hydrolysis and auto­(de)­phosphorylation in the soluble domains, with spatially distant transmembrane substrate translocation via a coupled actuator unit. Thus, we hypothesized that the LGL can produce new variants of sequences that preserve these multiple layers of complexity such as the ones present in functional P_1B‑1_-type ATPase transporters.

The LGL training input consists of a multiple sequence alignment (MSA) of the P_1B_-type ATPase family, comprising a refined set of approximately 13,500 sequences of 574–661 residue length (Figure S1A). This MSA was curated to minimize noise and sampling bias (see Methods). Figure 1A illustrates the methodology used to construct an LGL map of a total of 250,000 pixels, each representing a generated sequence with assigned Hamiltonian values. These generated sequences (pixels) can be decoded and analyzed in synergy with MD simulations, serving as a powerful integrated tool for protein design that effectively narrows the search space, enabling comprehensive characterization of the artificial transporters. When the P_1B_-type ATPase training data is plotted on the LGL in Figure 1B, a clustering pattern emerges, aligning with the established classification of the subgroups based on reported transmembrane motifs that correlate with transition metal cargo selectivities.^22^ Though the LGL allows us to potentially sample thousands of generated sequences within the subfamily, we narrowed down the selection to the P_1B‑1_ subgroup. This highly characterized subgroup, with available crystal structures, enables a robust and meaningful comparison and plays essential physiological roles across all organisms, including humans.^20,22,28^ Since P_1B‑1_ sequences from the training data set appear in the lower half of the map as purple symbols in Figure 1B, we hypothesize that the sequences decoded from this region are more likely to have P_1B‑1_-like functionality. With this in mind, we focused on the area that contained the well-characterized Cu­(I)-pump from *Legionella pneumophila* (*Lp*CopA) sequence for which structural information is available (PDB: 3RFU), thus serving as a reference. Figure 1C depicts a 3D representation of the region, where the red circle represents the location of WT *Lp*CopA sequence, and the nearby white circles, two decoded generated sequences GS1 and GS2 (selected out of eight characterized by MD, *vide infra*). Additionally, we ensured that the decoded sequences did not directly overlap with existing native sequences to increase the diversity of novel mutations (Figure S1B).

**Figure 1:**
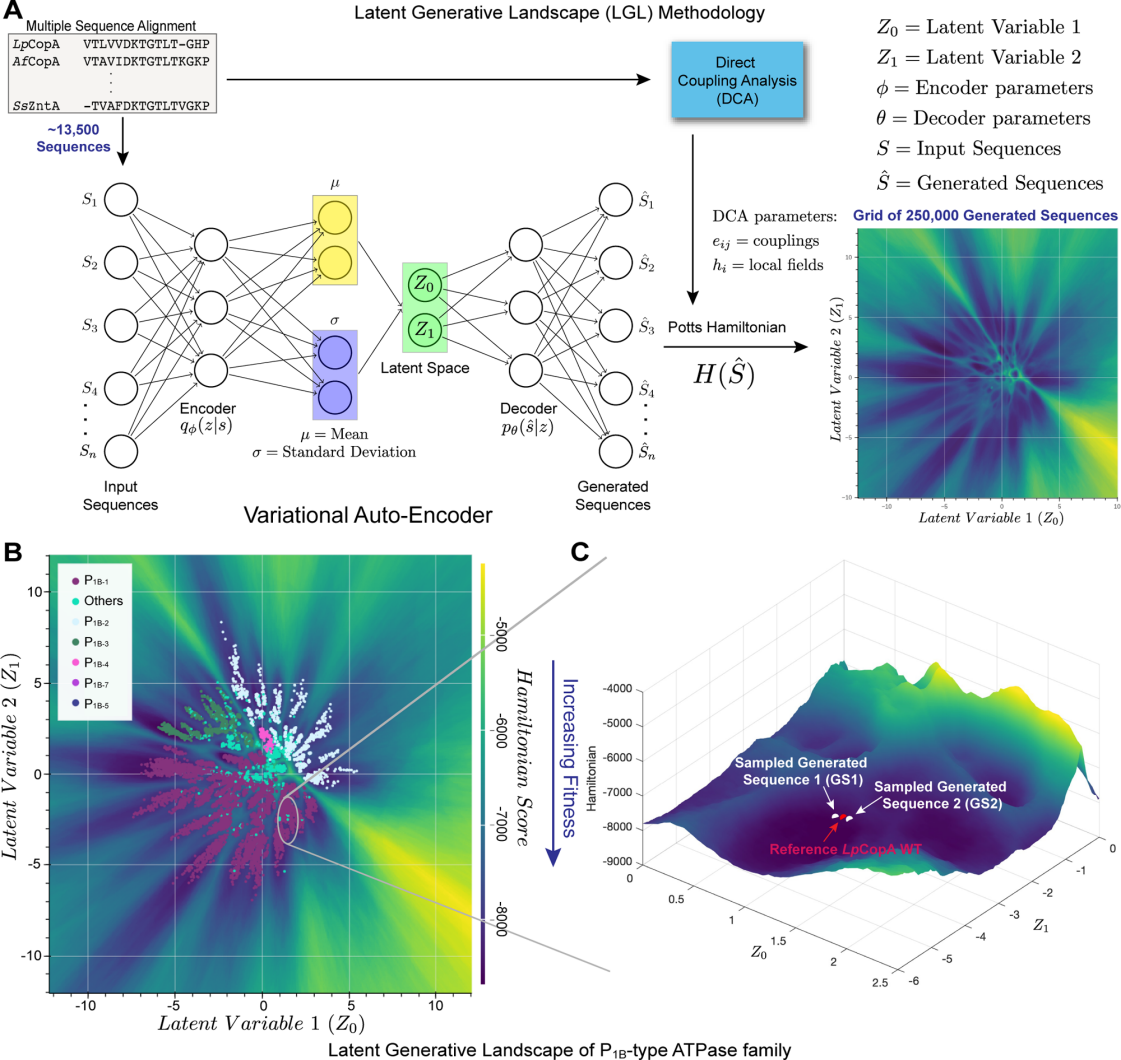
Overview of the Latent Generative Landscape (LGL) methodology. (**A**) The algorithm combines the generative power of the Variational Auto-Encoder (VAE) and the Potts Hamiltonian to approximate fitness of the generated sequences, resulting in an LGL map. (**B**) The LGL plot was generated using the assembled MSA for the P_1B_-type ATPase family. The labeled sequences from the training data set (13,553 sequences) are plotted on the LGL to identify the corresponding subgroup locations. (**C**) A 3D landscape view of the decoded region with reference *Lp*CopA labeled with a red circle, while the two selected decoded sequences (GS1 and GS2) are labeled in white.

### Structural Stability and Mutation Distribution of Generated Sequences

The domain topology diagram in Figure 2A illustrates that P_1B‑1_-type ATPases consist of a transmembrane (TM) domain featuring eight TM helices (MA-M6) responsible for metal substrate binding and forming the translocation pathway, three cytosolic domains (N-, nucleotide binding; P-, phosphorylation; A-, actuator), and metal-binding domain(s) (MBDs), located at either the N or C-terminal, serving a regulatory function.^29^ The deletion of MBDs has been shown to reduce the transport rate without affecting the transport mechanism or selectivity.^30,31^ Therefore, it was excluded from our LGL training data set and subsequent analysis.

**Figure 2:**
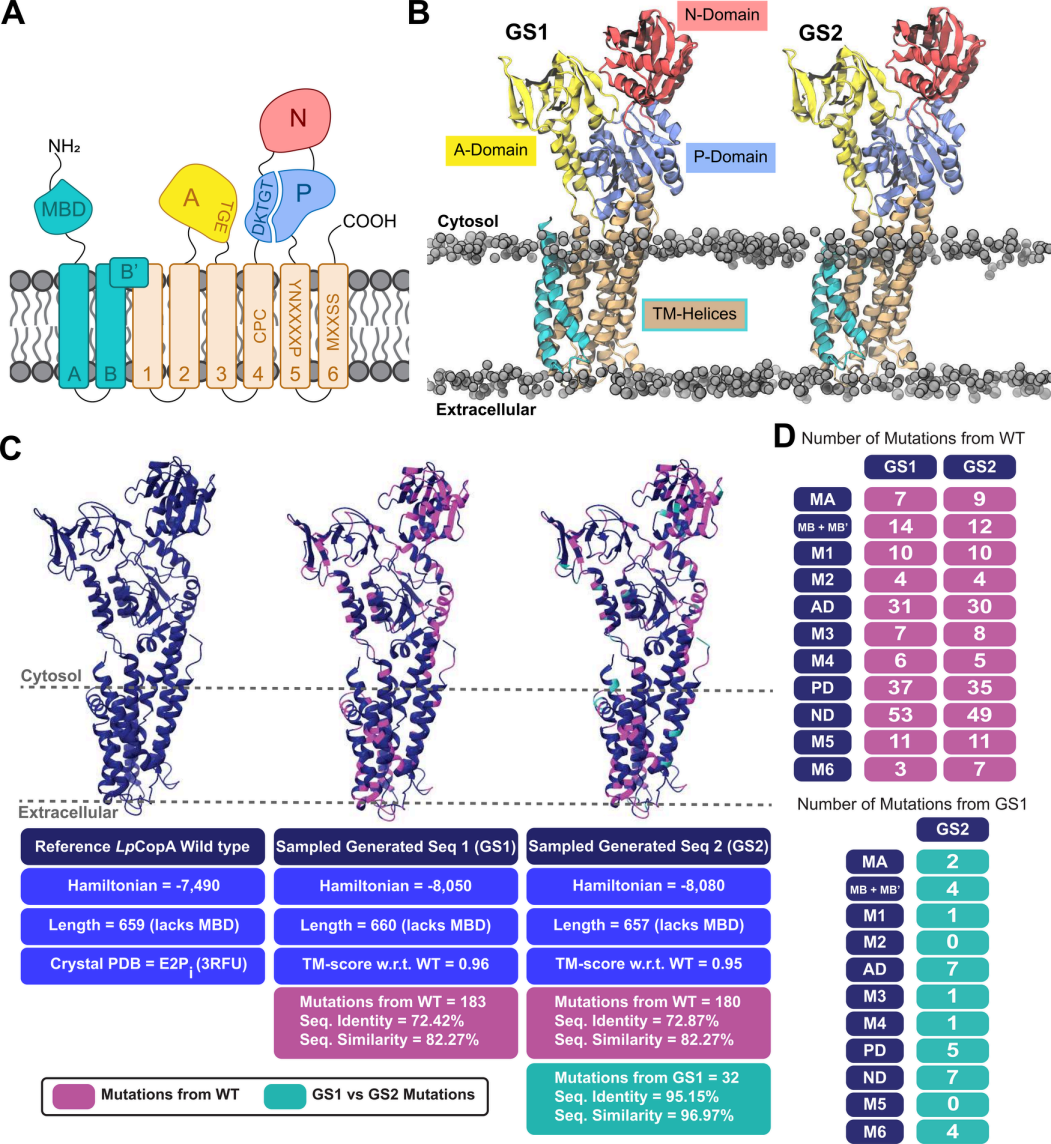
Structural and sequence composition details of the P_1B‑1_-type ATPase subgroup and the two selected decoded generated sequences. (**A**) A 2D domain topology of the P_1B_-type ATPase family with highlighted conserved motifs. DKTGT and TGE motifs on the P-domain and A-domain, respectively, are key in the auto­(de)­phosphorylation catalytic cycle characteristic of P-type ATPase transporters. Specific substrate selectivity motifs associated with the P_1B‑1_-type ATPase subgroup are labeled on M4, M5, and M6. (**B**) A 3D domain topology and membrane insertion of the generated proteins in lipid bilayers, prescreened via the PPM 2.0 server for membrane compatibility prior to MD simulation. (**C**) Comparison of structural, sequential, and fitness characteristics of GS1 and GS2 relative to WT *Lp*CopA. TM-score values indicate that the AF2-predicted structures closely match the crystal structure. Magenta highlights mutations relative to WT, while teal shows mutations relative to each other between the generated sequences. The Hamiltonian values infer that the generated sequences would maintain native-like functional fitness when compared to *Lp*CopA. (**D**) Domain-specific mutation count comparison for GS1 and GS2 versus *Lp*CopA in the top table, with the bottom table showing mutation counts between GS1 and GS2.

To better understand the distribution of the novel mutations within the described topological framework, Figure 2C highlights the mutation locations in the generated sequences, with corresponding labeled sequence alignments provided in Figure S2 and Figure S3. Notably, all critical conserved motifs were preserved with annotated locations in the sequence alignments, demonstrating the predictive power of the LGL. To explore the effect of these mutations on the 3D structure and stability, the sequences were first analyzed using TOPCONS to predict membrane topology, number and location of transmembrane helices, and delineate intracellular and extracellular domains.^32^ The predicted domain topology included eight TM helices and two large intracellular soluble regions that closely resemble that of the WT (Figure S4A). Consequently, AlphaFold2 (AF2) and Positioning of Proteins in Membranes 2.0 (PPM2.0) were employed to obtain structural and membrane insertion predictions for GS1 and GS2 presented in Figure 2B.^1,33^ The structures obtained showcase the characteristic eight TM helices and three catalytic soluble domains, as well as correct lipid bilayer insertion. The sequences had template modeling scores (TM-score) near 1 as reported in Figure 2C.^1,30^ This metric is commonly used in structural biology to quantify fold resemblance, with values ranging from 0 to 1, where 1 indicates a perfect match and an identical fold to the WT protein.^34^

Relative to *Lp*CopA, both GS1 and GS2 contained approximately 180 novel mutations, despite sharing 70% identity to the WT. These mutations were distributed throughout the protein where approximately 24% of the A-domain, 26% of the P-domain, 42% of the N-domain, and 23% of the TM region were mutated (Figure 2D). This result implies that the VAE provides each domain with flexibility in accommodating mutations without significantly disrupting the overall structure. A closer inspection revealed that both sequences contained a single insertion at V365 in the N-domain compared to the WT. This was notable, as removing this residue caused distortion of the first N-domain β-sheet (P359 to A367 in GS1 and P358 to A366 in GS2) in the AF2 model (Figure S4B). Furthermore, 400 ns MD simulations did not show early signs of β-sheet recovery (Figure S4C), although longer simulations may be needed to capture slower secondary structure changes. Interestingly, this insertion aligns appropriately with other P_1B‑1_ sequences (see position 397 in Figure S3), highlighting the predictive power of our generative model. Additionally, the generated sequences have 32 mutations among themselves with mutations scattered throughout the protein (Figure 2, C and D). Despite these variations, the Hamiltonian values reported for each sequence in Figure 2C are comparable, suggesting that these sequences maintain similar functional properties.

### Molecular Dynamics Identifies Crucial Dynamics of Generated Transporters

With high confidence in the structural integrity of the generated sequences, we performed MD simulations to investigate crucial dynamical properties. Structural characterizations of CopA in both eukaryotic and prokaryotic systems reveal a high degree of similarity (PDB: 3RFU, 8Q75, 7SI3, 4BBJ, and 8Q76 represent E2P/E2P_*i*_; PDB: 7R0I, 8Q74, and 7XUM represent E1-copper bound; PDB: 7R0G, 8Q73, and 7XUN represent E1-apo), indicating conservation of the Post–Albers mechanism cycle across the kingdoms (Figure 3A).^23,28,30,35–37^ This transport mechanism involves the intricate and coupled interplay of soluble domain movements driven by auto­(de)­phosphorylation events. These movements induce rearrangements within the TM domain, allowing the protein to alternate between two major states: the E1 inward-facing state, which has high affinity toward the substrate, and the E2 outward-facing state with lower affinity.^38^ Particularly, the E2P_*i*_ state is the starting point of a significant conformational change of the A-domain as the system evolves toward the E1 state.^30,35^ This A-domain movement triggers a substantial reorganization of the TM domain, visualized as two blocks of four TM helices each, (MA, MB, M1, M2) and (M3, M4, M5, M6), which move relative to each other, opening and closing the translocation pathways and making the binding site accessible to opposite sides of the lipid bilayer.^35^

**Figure 3:**
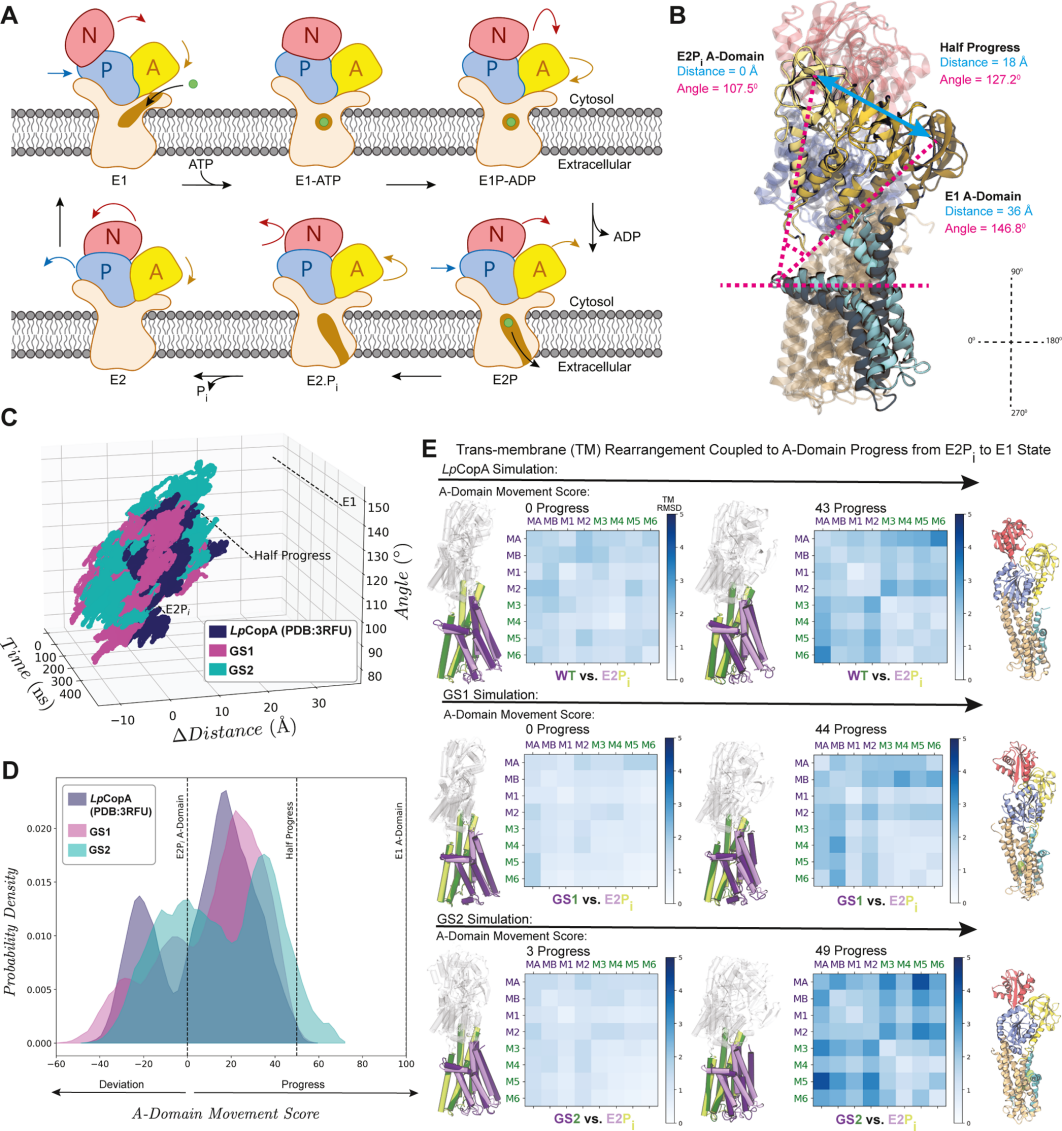
Molecular dynamics to capture key mechanisms and domain coupling required for metal substrate transport. (**A**) The P_1B_-type ATPase family follows the Post–Albers cycle. The pump converts between two states, E1 and E2, in an alternating access mechanism. Metal(s) bind to TM site(s) (E1 state), are occluded within the membrane upon ATP hydrolysis/phosphorylation (E1P), and are released on the opposite side in the E2P state, followed by a dephosphorylation transition state (E2P_*i*_) to then regenerate E1. During the E2P_*i*_ to the E1 transition, the A-domain undergoes a significant transition that is coupled to a rearrangement of the transmembrane transport pathway. (**B**) Structural alignment of the different stages of the A-domain transition along with labeled vectors used to calculate the tilt angle and Δdistance. (**C**) A-domain dynamics were measured over 400 ns simulations by tracking changes in tilt angle and Δdistance for *Lp*CopA, GS1, and GS2. (**D**) The *A-domain movement scores* for GS1 and GS2 show transitions similar to the WT. (**E**) Transmembrane helix rearrangement panels consist of structural alignments and TM helix distance difference matrices (DDM). Each matrix corresponds to the DDM observed at the specified *A-domain movement score*, with the value labeled above each matrix to indicate the progression toward the E1-like state. The purple block (MA–M2) and green block (M3–M6) represent the regions of the protein, with the E2P_*i*_ state shown in lighter tones and later A-domain stages in darker tones. The top row illustrates the rearrangement in the WT *Lp*CopA simulation followed by GS1 and GS2, respectively. The last column displays CAVER tunnels for each.

Our initial decoded list comprised eight generated sequences (GS1-GS8) with varying Hamiltonian values and locations on the LGL map (Figure 2C and Figure S5, A and D). To promote sequence diversity, we sampled from distinct regions within the same well, focusing on areas surrounding the *Lp*CopA region while capturing a broad range of Hamiltonian scores. Numerical Hamiltonian value alone, however, does not fully convey the extent to which interactions are retained or altered, nor their impact on the coupled dynamics. We aimed to explore various LGL regions around the *Lp*CopA area to gain a deeper understanding of the relationship between the calculated Hamiltonian and its impact on the system’s dynamics. Integrating MD simulations allowed us to elucidate intricate details regarding their A-domain and TM helix bundle rearrangement properties, enabling the evaluation of their consistency with the Post–Albers cycle and available crystal structures.

To assess the viability of the generated sequences as functional candidates, MD studies were conducted for *Lp*CopA and GS1-GS8. Each system underwent several 400 ns trials within an isobaric–isothermal (NPT) ensemble, with the protein embedded in a lipid bilayer and surrounded by a salt-containing aqueous solution. To exemplify the evaluation process, we will focus our discussion on the results of *Lp*CopA (PDB: 3RFU), GS1, and GS2, which were selected due to their native-like dynamics and favorable Hamiltonian values. Figure 3B illustrates the A-domain progression at different stages of the transition from E2P_*i*_ (PDB: 3RFU) to E1 (PDB: 7R0I) (outward- to inward-open) as well as the two metrics used for its movement quantification: the tilt angle (pink lines) and the Δdistance (blue lines). The distribution of these two values over the simulation time for the three systems is shown in Figure 3C, highlighting significant dynamics in the A-domain (see Movie S1). The angle and distance values were combined into a one-dimensional metric, termed the *A-domain movement score*, using equations S4-S7. Figure 3D shows that positive values, defined as “progress”, correspond to A-domain motions toward the E1 state, while negative values, referred to as “deviation”, indicate motions in the opposite direction. Although WT *Lp*CopA did not fully transition to the E1 state due to the limitations associated with capturing large conformational changes using all-atom simulations, the overlapping bimodal distribution among the three proteins illustrates similar A-domain dynamics to those of the WT, indicating comparable coupled domain movements characteristic of the Post–Albers catalytic cycle. The results for each system trial are detailed in Figure S6, A and C.

Figure 3E summarizes the qualitative and quantitative analyses used to assess the TM interblock rearrangement. The superposition of structures, with purple representing the first TM block and green the second block, was combined with the helix Distance Difference Matrix (DDM) to observe and quantify the rearrangement. Results from the *Lp*CopA simulations (Figure 3E *top* panel), show a pattern clearly mimicking the proper interblock distance changes observed in experimentally determined crystal structures in Figure S6B.^23,30,35^ In the DDM for the 100% A-domain progress in Figure S6B, a large interblock rearrangement is observed at the bottom-left and top-right corners, while the intrablock rearrangement showed only small RMSD values. We demonstrated that, as the A-domain of both generated sequences transitions from the E2P_*i*_ to the E1 state, the TM block movement begins to resemble that of a coupled native transporter, with GS2 showing this more clearly (Figure 3E *bottom* panel, Movie S2). GS2 also starts to exhibit an E1-like transmembrane tunnel, as indicated by CAVER 3.0 analysis (Figure 3E *right-most bottom* panel).^39^ These results suggest that the modeled systems retain the essential mechanistic features required for their biological activity, revealing dynamic properties that complement the LGL approach.

The A-domain and corresponding helix bundle movement rearrangement analyses were also carried out for the GS3-GS8 sequences (Figure S5B-D; Tables S1-S2; see Supplemental for detailed analysis). Applying MD simulations to these variants enabled us to directly link learned evolutionary constraints to their conformational dynamics. Synchronizing LGL predictions with MD simulations allowed us to identify which aspects of these dynamics in the decoded eight sequences were impacted and to what extent, yielding mechanistic insights complementary to the Hamiltonian scores. Our criteria for experimental characterization prioritized variants that exhibited the best coordinated movement of both the A-domain and the transmembrane (TM) bundle. Notably, GS1 and GS2 demonstrated superior performance in preserving both of these desired dynamic properties, while the other variants typically captured one of these motions, but not both. Thus, GS1 and GS2 were subjected to experimental characterization.

### Experimental Characterization of Preserved WT Functionality

The two selected generated sequences were subjected to experimental characterization to validate membrane insertion, correct folding, *in vitro* catalytic ATP hydrolysis activity, and *in vivo* cellular protection from copper toxicity underlying transport capability. TM domain folding was assessed by analyzing membrane localization upon recombinant expression in *E. coli* membrane fraction (MF), while folding of the soluble domains was validated through *in vitro* ATP hydrolysis activity, ensuring the protein retained its ATP hydrolysis and P-domain’s autophosphorylation turnover. Copper transport activity was evaluated *in vivo* by testing recombinant GS1 and GS2 expression via intracellular copper quantification and protection from copper toxicity.

Initial results, presented in the Western blot in Figure 4A, confirmed successful recombinant expression despite each generated protein featuring mutations in approximately one-third of its sequence. Both protein constructs were primarily localized to the MF, indicating proper insertion in the lipid bilayer and TM domain folding. This is supported by the significantly monodisperse Size Exclusion Chromatography (SEC) profiles upon extraction and purification in detergent micelles (Figure S7A). No significant misfolded proteins were indeed observed in the soluble fraction (SF) or inclusion bodies.

**Figure 4:**
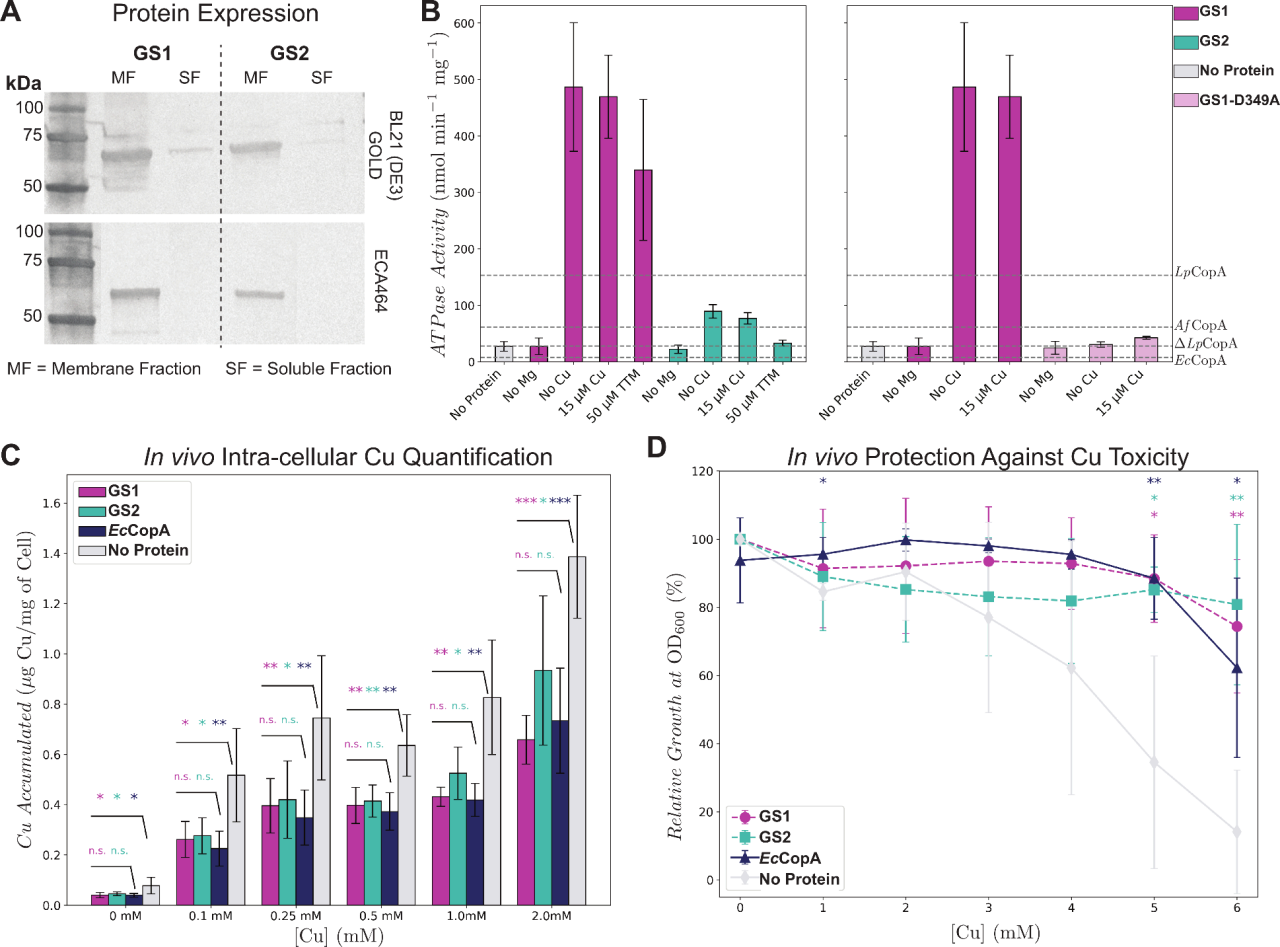
Experimental characterization of expression, membrane embedding, hydrolysis activity, and copper export for GS1 and GS2. (**A**) Western blot analysis reveals successful protein expression and insertion of both GS1 and GS2 in the membrane fraction. (**B**) *In vitro* ATPase activity rate for GS1 and GS2 in comparison to several characterized P_1B‑1_-type ATPase transporters (dashed line). WT Δ*Lp*CopA lacks the MBD, providing an ideal comparison with GS1 and GS2. GS2 exhibited rates that are in range with the reported values for characterized prokaryotic P_1B-1_-type ATPases, while GS1 showed a significantly higher catalytic rate.^21,30,40^ The GS1-D349A mutation, which abolished ATP hydrolysis activity, confirmed that the defining auto­(de)­phosphorylation of the aspartate in the conserved DKTGT motif in the P-domain was preserved. (**C**) *In vivo* efflux assay demonstrates that both GS1 and GS2 can effectively export copper from cells as a function of copper concentrations, similar to WT *Ec*CopA, while the absence of a transporter leads to metal accumulation within the cells. (**D**) *In vivo* copper-susceptibility growth assay confirms that both GS1 and GS2 can protect cells against copper toxicity and promote cell viability as a function of increasing copper concentration, similar to overexpression of WT *Ec*CopA. Statistical significance was calculated by unpaired Student’s *t*-test (*** for *p* < 0.001, ** for *p* < 0.01, and * for *p* < 0.05; n.s. for not significant).

Upon successful purification in detergent micelles (Figure S7A), we determined the specific ATP hydrolysis activity rates as this is a defining catalytic biochemical property of all P-type ATPases. Using *in vitro* malachite green assays, which allow colorimetric quantification of released inorganic phosphate upon ATP hydrolysis, we demonstrated that both GS1 and GS2 exhibit high catalytic turnover rates, despite lacking the MBD. As shown in Figure 4B, ATPase rate of GS2 (77 *nmol* min^–1^ *mg* ^–1^) is comparable to characterized native P_1B‑1_-type ATPases (dashed lines) while GS1 (469 *nmol* min^–1^ *mg* ^–1^) exceeded those values.^21,30,40^ This raised the question of whether the mutations may have compromised the transporter’s ability to autophosphorylate at the conserved DKTGT motif present in the P-domain. To ensure that ATP hydrolysis remained coupled to P-domain phosphorylation, we generated GS1-D349A, lacking the conserved aspartate residue, which undergoes the required phosphorylation essential to complete the Post–Albers cycle. The absence of any ATPase catalytic activity in GS1-D349A confirmed that ATP hydrolysis in the generated sequence is strictly coupled and dependent on autophosphorylation (Figure 4B). This result can be correlated to the minor differences observed in transmembrane domain RMSD during the simulations (Figure S6A) in which the WT protein displays a more rigid behavior, suggesting reduced interdomain coupling dynamics that could lead to lower activity efficiencies. However, while the ATPase activity in native characterized P_1B‑1_-type ATPase is strictly dependent on the presence of Cu­(I) substrates, our *in vitro* assay did not show the expected copper dependency. We proposed that this behavior resulted from the μM level copurification of copper with the protein, suggesting a very high affinity of the generated constructs toward Cu­(I). This was further supported by the observed reduction but incomplete abolishment of ATP hydrolysis activity upon treatment with up to 50 μM tetrathiomolybdate (TTM), a copper chelator with K_*d*_ of 2.32 × 10^–20^ M,^41^ which effectively competes with Cu­(I) availability with the Cu­(I)-pumps (Figure 4B).

Thus, to unambiguously confirm functionality *in vivo*, copper susceptibility and metal quantification assays were performed upon expression of GS1 and GS2 in native and copper-sensitive *E. coli* strains. First, the ability of GS1 and GS2 to efficiently extrude Cu­(I) from the cytosol was investigated via *in vivo* intracellular copper quantification by Inductively-Coupled Plasma Mass Spectrometry (ICP-MS) upon cellular growth in media containing increasing copper concentrations. In this assay, we expected functional copper exporters to reduce intracellular copper accumulation. *E. coli* cells that were recombinantly expressing GS1 and GS2 showed reduced copper concentrations compared to cells transformed with a control plasmid not encoding for any Cu­(I) P-type ATPase pump (Figure 4C). In agreement with native-like function, GS1 and GS2’s ability to reduce cellular concentration aligned well with cells overexpressing the functional native *Ec*CopA pump. To further validate the ability of GS1 and GS2 to protect cells from copper toxicity by extrusion, *in vivo* copper metal susceptibility assays were performed in the copper-sensitive *E. coli* ECA464 strain lacking the endogenous bacterial copper efflux and detoxification system genes (*copA, cueO, and cusCFBA*).^42^ In this assay, protection from copper toxicity was determined by monitoring bacterial growth under increasing copper concentrations to evaluate the protective effect of the expressed transporters. As shown in Figure 4D (with detailed OD_600_ data from a representative trial in Figure S7B), at a concentration of 6.0 mM copper, cells expressing GS1 (74.6% mean-normalized relative growth) and GS2 (80.8%) demonstrated comparable or exceeding protection ability against copper toxicity than cells expressing the native *Ec*CopA (62.3%). Contrarily, cells transformed with a corresponding empty control vector (14.1%) featured significant growth inhibition due to copper toxicity at higher copper concentrations. Together, experimental *in vitro* and *in vivo* characterization indicate that both generated sequences retain the expected functionality of a P_1B‑1_-type ATPase, effectively exporting copper from the cellular cytosol.

## Conclusion

Cu­(I) transmembrane pumps are complex ATP-dependent multidomain transporters responsible for maintaining essential metal homeostasis in cells in all kingdoms of life. Designing membrane proteins of this level of complexity is significantly challenging because it requires addressing intricate structural dynamics, achieving stability in lipid bilayers, and assessing their functional ability in a native-like environment. Through synergy of machine learning, molecular dynamics, and experimental validation, we designed two artificial transmembrane Cu­(I) P-type ATPase pumps (GS1 and GS2), each containing approximately 180 mutations from the referenced WT *Lp*CopA sequence. The LGL framework successfully decoded nonextant novel variants by leveraging preserved coevolutionary interactions, learned exclusively from the sequence space. The integration of MD and experimentation allowed characterization of essential dynamics and function, providing insights into structural and functional behaviors expected to be preserved in the generated sequences. Results showed that both were capable of adopting the correct fold required for membrane insertion, maintained coupled domain movements, and provided protection against copper toxicity through export across the membrane. The functional efficacy of these artificial transporters marks a major step forward in the field of evolutionary-inspired membrane protein design, offering new possibilities for future advancements such as optimization of protein function efficiency or design of *de novo* protein folds.

The interdisciplinary approach presented here can also be applied to other complex, less characterized systems to advance our understanding and engineering of biological systems. Our learning framework integrates an in-depth exploration of sequence space, dynamics, and the biochemical properties of the protein system under consideration. This has significantly narrowed the gap between design predictions and experimental characterization, a trend likely to continue as we deepen our understanding of the functional patterns within protein families as a whole. The potential applications are vast, offering the promise of inspiring numerous additional approaches aimed at designing protein functions in a logical and time-efficient manner. Indeed, one of the most immediate and impactful applications of this workflow could be optimization of membrane protein stability and function. Provided a sufficiently deep MSA is available, our synergistic pipeline remains applicable, as the LGL model functions purely at the sequence level and does not depend on structural input to generate new variants. While a modeled 3D structure or functional intuition can enhance the structural and dynamical analyses, it is not strictly necessary. When applied to AlphaFold predicted or template-based models, molecular dynamics simulations can still reveal differences in dynamic behavior relative to a wild-type reference, without the need for predefined collective variables. This flexibility makes the workflow adaptable and could enable the generation of more stable homologues, thereby facilitating structural and functional characterization of otherwise intractable membrane proteins. We acknowledge, however, that in systems lacking detailed mechanistic insight, interpreting the functional relevance of the observed dynamics may remain challenging.

An important avenue for further investigation includes the analysis of the 32 mutations distinguishing GS1 and GS2 as a potential means of controlling ATP hydrolysis rate. This energy transduction process drives the conformational changes necessary for ion transport. Investigating how these mutations modulate ATP hydrolysis could provide insights into the energy transduction mechanism and overall catalytic efficiency in transmembrane transition metal pumps, and P-type ATPases at large. In addition, designing novel transmembrane proteins with altered substrate specificity offers a powerful approach to understanding the flexibility of the sequence space and the evolutionary limits of variability within this family. This established framework now sets the stage for future efforts aimed at modifying substrate specificity or tuning transport kinetics. From an application standpoint, designed variants could also be engineered for increased thermostability or detergent compatibility, facilitating purification and experimental characterization.

## Materials and Methods

No unexpected or unusually high safety hazards were encountered.

### HMMER and HMMSearch for MSA Generation

We used the HMMER software package to identify homologous protein and nucleotide sequences. The *HMMSearch* tool generates a multiple sequence alignment (MSA) based on a profile Hidden Markov Model (HMM) that represents patterns associated with a sequence family.^43^ The initial profile HMM is created on the basis of either a sequence or a small MSA consisting of sequences that belong to the family of interest. Databases are searched to identify additional homologous sequences that are appended to the initial MSA and update the statistical model accordingly. After this iterative procedure reaches a threshold, *HMMSearch* stops identifying additional sequences, and a final MSA is obtained.

### MSA of the P_1B_-Type ATPase Family

In the CopA system, the metal binding domains (MBDs) have experimentally been demonstrated to only influence the rate of transport but not cargo selectivity and overall translocation abilities, and were thus excluded from the training set.^20^ Furthermore, the inclusion of this domain reduced the resulting number of sequences and diversity in the MSA because it is only associated with certain subgroups.^22^ Using the remaining sequence portions, P_1B‑1_ *Legionella pneumophila* CopA (*Lp*CopA) and P_1B‑2_ *Shigella sonnei* ZntA (*Ss*ZntA) were aligned to create the initial profile HMM for *HMMSearch*. The master data set was refined by subsampling sequences with below 87% identity and limiting gaps to 25 continuous positions to reduce redundancy and noise. The resulting MSA comprised 13,553 sequences, with full distribution details provided in Figure S1A. This data set served as input for training the LGL map shown in Figure 1B.

### Direct Coupling Analysis (DCA)

Direct coupling analysis (DCA) is a statistical method that infers direct residue–residue interactions and contacts in the 3D fold of a protein family based on the identification of coevolving residues learned from an MSA.^44^ Each predicted residue–residue interaction is assigned a score called the Direct Information (DI) score, where a higher value indicates more coupled interaction between an amino acid pair. These direct pairs are identified using a Potts model, which quantifies interaction strengths between residues by incorporating both single-site local field parameters *h*_*i*_(*s*_*i*_) and pairwise coupling parameters *e*_*ij*_(*s*_*i*_, *s*_*j*_) trained on the input MSA.^45^ The parameters *e*_*ij*_(*s*_*i*_, *s*_*j*_) quantify the coupling strength between the positions of the residues *i* and *j* for all possible pairs of amino acids. Strong coupling pairs represent the likelihood of coevolving relations among residue positions beyond chance. The *h*_*i*_(*s*_*i*_) parameters capture the amino acid biases for independent positions, a measure of conservation.

The above parameters can be estimated to derive the Potts model equation below, in which P(Ŝ) represents the probability that the specific sequence Ŝ belongs to the family represented by the MSA.^44^

1 $$P\left(Ŝ\right)=\frac{1}{Z}\exp\left(\underset{i}{\sum }h_{i}\left(s_{i}\right)+\underset{i<j}{\sum }e_{ij}\left(s_{i},s_{j}\right)\right)$$

A Hamiltonian (*H*) equation can be derived from this Potts model, which contains the set of parameters that model the “sequence energy” of the system:^44,46^

2 $$H\left(Ŝ\right)=-\underset{i}{\sum }h_{i}\left(s_{i}\right)-\underset{i<j}{\sum }e_{ij}\left(s_{i},s_{j}\right)$$

In the context of sequences, this Potts Hamiltonian equation measures the fitness of the specific sequence *Ŝ* by taking the sum of the *e*_*ij*_(*s*_*i*_, *s*_*j*_) and *h*_*i*_(*s*_*i*_) parameters determined from the input MSA of the protein family. A greater negative Hamiltonian value predicts relatively higher functional fitness compared to a greater positive value.^46,47^

### Variational Auto-Encoder (VAE)

The Variational Auto-Encoder (VAE) is an unsupervised generative machine learning architecture that compresses high dimensional data into a reduced set of latent variables (*z*). The architecture consists of three parts: 1) encoder, 2) latent space, and 3) decoder. The model is trained upon input data*S*, which is fed through the encoder network, *q*_ϕ_(*z*|*S*), to learn the features and associated weights that represent the data. Based on these learned parameters, an approximate Gaussian probability distribution is derived to represent the compressed latent space defined by the specified latent variables (*z*). Each latent variable in the latent space is a unique combination of essential features and random noise that represents novel variants of the input data set. Each set of *z*-variables (*z*_0_, *z*_1_) from this latent space distribution is fed through the decoder network $p_{\theta }\left(Ŝ|z\right)$ , producing the reconstructed data *Ŝ*.

The overall objective of the architecture is to maximize the evidence lower bound (ELBO) function below:^48^

$${ELBO}_{VAE}\left(\theta ,\phi ;S\right)=E_{q_{\phi }\left(z|S\right)}\left[\log p_{\theta }\left(Ŝ|z\right)\right]-KL\left[q_{\phi }\left(z|S\right)∥p_{\theta }\left(z\right)\right]$$ 3

In the ELBO, the first term $E_{q_{\phi }\left(z|S\right)}\left[\log p_{\theta }\left(Ŝ|z\right)\right]$ measures the reconstruction quality, while the second term KL­[*q*_ϕ_(*z*|*S*)∥*p*_*θ*_(*z*)] is the regularization. The Kullback–Leibler (KL) divergence encourages the learned encoder distribution *q*_ϕ_(*z*|*S*) to follow the prior distribution *p*_*θ*_(*z*) that is set as a standard Gaussian, thus resulting in the approximate Gaussian probability distribution representative of the latent space.

### Latent Generative Landscape (LGL) Framework

We implemented the Latent Generative Landscape (LGL) architecture, which is an enhanced framework of the VAE that incorporates fitness metrics based on amino acid coevolution, to study protein systems.^14^ The DCA model generates the *e*_*ij*_(*s*_*i*_, *s*_*j*_) and *h*_*i*_(*s*_*i*_) parameters, while the VAE reconstructs sequences *Ŝ* using the same MSA input *S*. Each reconstructed sequence is assigned a fitness score H(Ŝ) based on the DCA parameters, and the final output is the LGL map (Figure 1A). When visualized in 3D, this map resembles a topographic view, where different colors, valleys, and peaks represent relatively favorable or unfavorable energy for the generated sequences (Figure 1C).

To visualize the dynamics of fitness across the protein family, a 500 × 500 coordinate grid LGL is produced using a curated MSA as input as demonstrated in Figure 1A *right*. Each pixel in the map represents a reconstructed sequence *Ŝ* from the VAE. Each reconstructed sequence is scored based on its Hamiltonian value H(Ŝ) with respect to the family and is color-coded accordingly.^44,46^ The dark native blue valleys represent low-energy, favorable configurations while the bright green peaks indicate sequences less explored by the extant data set.

Overlaying native input sequences onto the generated LGL map provides deeper insights into phylogenetic relationships and the distribution of features across the latent space.^2,13,14^ Figure 1B presents the LGL of the P_1B_-type ATPase family, with colored symbols representing labeled sequences from the input training MSA, categorized by their corresponding subgroups. Sequences within valleys or clusters share common characteristics, allowing a chance to explore underlying patterns previously unidentified within a protein family.^3^ Novel reconstructed sequences *Ŝ* can be decoded based on fitness score and shared characteristics with overlapping native family sequences *S*.

### Hyperparameters and Training of the LGL Model

All hyperparameters from Ziegler *et al*. were adopted to train the LGL model for this system. Specifically, the model used 3 × *L* hidden units in both the encoder and decoder, where *L* is the length of the sequences in the MSA (*L* = 663 in this case). ReLU activation functions were applied to the hidden units. The latent dimension was set to 2 (*z*_0_, *z*_1_), and the model was optimized using the Adam optimizer with a learning rate of 1 × 10^–4^ and an L2-regularization penalty of 1 × 10^–4^ applied to the hidden units. Training was terminated when the loss failed to improve for 10 consecutive epochs.

### Molecular Dynamics System Preparation

The starting structures (*Lp*CopA, GS1-GS8) were generated by homology modeling with the E2P_*i*_ template structure (PDB: 3RFU) using SWISS-MODEL.^49,50^ Afterward, protonation states at pH 7.4 were assigned using the H++ online server.^51–53^ Additionally, protein orientations within the lipid bilayer were determined using the Positioning of Proteins in Membranes (PPM2.0) algorithm.^33^ Subsequently, each system was prepared for MD simulations using the Amber20 suite.^54^ This involved using the *tleap* program to cap the termini accordingly, while *packmol-memgen* embedded the proteins in a lipid bilayer with a composition of six 1-palmitoyl-2-oleoyl-sn-glycero-3-phosphoethanolamine (POPE), three 1-palmitoyl-2-oleoyl-sn-glycero-3-phosphocholine (POPC), two 1-Palmitoyl-2-oleoyl-sn-glycero-3-phosphoglycero (POPG), and one 1,3-bis­(1-oleoyl-2-palmitoyl-sn-glycero-3-phospho)-sn-glycerol (OPOPCL). The systems were solvated with water and Na^+^ ions were added to neutralize the negatively charged phospholipid heads.^54–56^ Then, *tleap* was used again to introduce a 150 mM NaCl concentration atop the neutralizing ions and to generate the parameter and coordinate files. The force fields used included ff14SB, lipid17, lipid17*ext*, and TIP3P for water.^57–60^

### Molecular Dynamics Simulation Protocol

MD simulations were conducted using the GPU-accelerated *pmemd* program within the Amber20 suite.^54^ Initial energy minimization was carried out in four stages, each consisting of 5000 steps of steepest descent followed by 5000 steps of conjugate-gradient minimization, progressively reducing the amount of restraints. Restraints of 20 kcal/mol/Å^2^ were applied as follows: first, on all atoms except lipids; then to protein and water atoms; next, exclusively to protein atoms; and, finally, with no restraints. The systems were heated in two phases: from 0 to 100 K over 6 ns, and from 100 K to 303 K over 12 ns, using a Langevin thermostat while maintaining the lipid bilayer and protein under soft restraints (5 kcal/mol/Å^2^).^61^ After heating, a membrane equilibration protocol was applied with ten steps of 500 ps to stabilize the periodic boundary conditions. Subsequently, production of MD simulations was run in trials of 400 ns, each under the NPT ensemble with a 2 fs time-step, Langevin dynamics, a 12 Å cutoff for nonbonded interactions, and the SHAKE algorithm.^62^ System stability and convergence were assessed by monitoring the RMSD and conducting unit root tests on each trial to verify the attainment of a stationary state, as illustrated in Figure S6A and detailed in table S3, respectively. Unit root test results indicated that all trials reached a stationary state. Data analysis was performed after exclusion of first 50 ns of each trial to accommodate for equilibration using the *cpptraj* module in Amber20 and Tcl script for VMD.^54,63,64^

### A-Domain Conformational Analysis

The transmembrane helices in each trajectory were aligned with those in the initial structure of the E2P_*i*_ state of *Lp*CopA (PDB: 3RFU) to ensure consistency and accurate measurement. Two metrics were computed using an in-house Tcl script for VMD to capture the movement of the A domain: the tilt angle and the displacement distance denoted as Δdistance. The tilt angle was calculated by taking the cross product of two orthogonal vectors, which are highlighted by the pink dashed lines in Figure 3B. The first vector, representing a stable horizontal reference perpendicular to the membrane normal vector, was defined along the MB’ helix kink at the lipid bilayer-solvent interface. The second vector extended from one of the basal α-helices to the center of mass of the A-domain’s β-sheets, providing a direct measure of domain tilt relative to the membrane plane. The second metric, Δdistance, was defined as the distance between the center of mass of the A-domain in each frame and its center of mass in the E1 state (PDB: 7R0I), depicted as the blue solid line in Figure 3B. The Δdistance allowed us to evaluate any translational movement of the domain toward the E1 state. *Lp*CopA and GS1-GS8 results for each trial are presented in Figure S5C and S6C. To streamline interpretation, these metrics were combined into an *A-domain movement score* as follows:

1. Frame vector construction: the frames’ angle and displacement distance scalar values (θ and Δd) were combined to create 2D vectors.

4 $$Frame\;vector=F_{j}=\left(F_{j\theta },F_{j\Delta d}\right)$$

2. Frame Euclidean distance: for each frame, the Euclidean distance between the frame vector and the target vector (E1) was calculated as:

5 $$Frame\;Euclidean\;distance=∥E1-F_{j}∥=\sqrt{{\left(E1_{\theta }-F_{j\theta }\right)}^{2}+{\left(E1_{\Delta d}-F_{j\Delta d}\right)}^{2}}$$

3. Target Euclidean distance: the Euclidean distance between the reference vector (E2P_*i*_) and the target vector (E1) was calculated as shown:

6 $$Target\;Euclidean\;distance=∥E1-E2P_{i}∥=\sqrt{{\left(E1_{\theta }-E2P_{i\theta }\right)}^{2}+{\left(E1_{\Delta d}-E2P_{i\Delta d}\right)}^{2}}$$

4. Movement score calculation: the frame Euclidean distance was compared to the target Euclidean distance to calculate the *A-domain movement score* for each frame:

7 $$A\mathrm{‐d}omain\;movement\;score=\left(1-\frac{∥E1-F_{j}∥}{∥E1-E2P_{i}∥}\right)\times 100$$

The resulting probability density of the *A-domain movement scores* provides a comprehensive measure of A-domain conformational change (Figure 3D and Figure S5B). Positive values signify movement toward the E1 state and are labeled as “progress,” while negative values indicate movement away from the E1 state and are labeled as “deviation.” Additionally, table S1 summarizes the maximum coupled A-domain progress achieved in each sequence trial.

### Transmembrane Helix Rearrangement Analysis

A-domain rearrangement is functionally meaningful only if it induces conformational changes in the transmembrane (TM) domain. In P_1B‑1_-type ATPases, these conformational changes involve the relative movement of two distinct blocks, each containing four transmembrane α-helices (MA, MB, M1, M2) and (M3, M4, M5, M6). To quantify this movement, we performed both qualitative and quantitative analyses. For qualitative analysis, we visualized the superposition of the two TM blocks (colored green and purple) at various stages of the A-domain progress, relative to the reference starting structure of *Lp*CopA (PDB: 3RFU), depicted in lighter shades of the same colors in Figure 3E. This visualization provided an intuitive representation of the directional shifts in TM blocks over time. For quantitative analysis, we utilized the *pyDDM* tool to compute a distance difference matrix (DDM), enabling detailed measurement of both intrablock and interblock changes across trajectory frames relative to the reference structure.^65^ This matrix allowed us to detect interblock rearrangements with high precision with table S2 showcasing the maximum coupled interblock DDM values featured in each sequence trial. The combination of quantitative distance analysis and qualitative visualization provided a comprehensive picture of TM block movement and confirmed whether the block shifts remained aligned with the *A-domain movement score*. Additionally, CAVER 3.0 analysis was also performed to monitor binding site accessibility on both the intracellular and extracellular sides adding another layer of verification for TM helix rearrangement. According to the Post–Albers cycle, simulations exhibiting large A-domain transitions are expected to show transmembrane rearrangements that occlude the release pathway while subsequently exposing the uptake pathway, as shown in Figure 3E.

### Protein Expression of Selected Generated Sequences

The codon optimized synthetic DNA encoding GS1 and GS2 sequences (Genscript Inc.) was cloned into a pET-52b­(+) vector with an N-terminal STREP-tag II and a C-terminal His-Tag. The constructs were then transformed into *E. coli* BL21-Gold­(DE3) competent cells for recombinant protein expression. Transformed cells were grown aerobically overnight in Terrific Broth (TB) media supplemented with 50 μg/mL ampicillin. The overnight cultures were subsequently inoculated into fresh TB media containing ampicillin (50 μg/mL) and grown aerobically at 37 °C with shaking (240 rpm) until they reached OD_600_ 2.0. Protein expression was induced by the addition of 0.5 mM IPTG and cells were incubated at 30 °C for an additional 6 h. Cells were harvested by centrifugation at 4,000 x*g* for 20 min at 4 °C.

Harvested cells were resuspended in lysis buffer (20 mM Tris-HCl pH 8, 150 mM NaCl, 5 mM MgCl_2_, 30 μg/mL DNaseI from bovine pancreas, and EDTA-free protease inhibitor cocktail (Thermo Scientific)), and lysed using an ice-cooled microfluidizer at 20,000 psi for four cycles (Microfluidics M-110P). The lysate was subjected to two centrifugation steps: first at 20,000 x*g* for 20 min at 4 °C to remove cell debris and subsequently at 180,000 x*g* for 1 h at 4 °C to pellet the cell membranes. The membrane pellet was resuspended in buffer (20 mM Tris-HCl pH 8, 500 mM NaCl, 10% (w/v) glycerol, and EDTA-free protease inhibitor cocktail (Thermo Scientific)), flash-frozen in liquid nitrogen, and stored at −80 °C until purification. Also, to confirm protein expression, the resuspended membranes and supernatant from the last centrifugation were subjected to SDS-PAGE and Western Blot. Samples containing approximately 15 μg of total protein were subjected to SDS-PAGE (4–15%). Consequently, protein bands were turbo-transferred onto a nitrocellulose membrane, which was immediately blocked using 3% nonfat milk in TBST buffer (Tris Buffered Saline buffer with a 0.1% concentration of Tween 20) at room temperature for 1 h. Then, the primary antibody against STREP-tag II (Thermo Scientific, MA542540) was added in 3% milk in TBST onto the membrane and incubated overnight at 4 °C. Afterward, the membrane was rinsed with TBST buffer, and the alkaline-phosphatase conjugated secondary antimouse IgG antibody (Invitrogen, A16069) in 3% milk in TBST buffer was poured onto the membranes for 2 h at room temperature. Lastly, after rinsing the membranes with TBST the bands were developed using nitroblue tetrazolium (NBT) and 5-bromo- 4-chloro-3-indolyl phosphate (BCIP) in Tris buffer.

### Protein Purification of Selected Generated Sequences

Purification followed an established protocol for *Ec*CopA.^21^ Briefly, membrane proteins were extracted by incubating the membrane suspension with 1% (w/v) n-dodecyl-β-d-maltoside (DDM) detergent in buffer (20 mM Tris-HCl pH 8, 500 mM NaCl, 25 mM imidazole, 5 mM β-mercaptoethanol and EDTA-free protease inhibitor cocktail) for 1 h at 4 °C, followed by ultracentrifugation at 180,000 x*g* for 30 min at 4 °C to remove residual membranes. The detergent-solubilized supernatant was loaded onto a 5 mL HisTrap affinity column (Cytiva) at 0.5 mL/min flow rate in binding buffer (20 mM Tris-HCl pH 8, 500 mM NaCl, 25 mM imidazole, 1 mM DTT, and 0.05% (w/v) DDM) followed by extensive washing of the column using the same buffer for 40 column volumes. Protein elution was achieved with elution buffer (20 mM Tris-HCl pH 8, 500 mM NaCl, 400 mM imidazole, 1 mM DTT, and 0.05% (w/v) DDM). The eluted protein was loaded onto a HiPrep 26/10 desalting column (Cytiva) equilibrated with 20 mM MOPS/HCl pH 7, 500 mM NaCl, 1 mM EDTA, 1 mM DTT, and 0.05% (w/v) DDM to remove imidazole and exchange the buffer. The protein was concentrated using a 100 kDa MWCO spin concentrator to 2 mg/mL and afterward loaded onto a Superdex S200 10/300 size exclusion chromatography column (Cytiva) for isolation of monodisperse protein using sizing buffer (20 mM MOPS/HCl pH 7, 500 mM NaCl, 1 mM DTT, and 0.05% (w/v) DDM). SDS-PAGE (4–15%) confirmed purity of the proteins, and concentration was determined by Abs_280_ (GS1 MW = 71.6 KDa and *ε*_280_=58,440 *M* ^–1^ *cm* ^–1^, GS2 MW = 70.4 KDa and *ε*_280_=56,950 *M* ^–1^ *cm* ^–1^). The purified protein samples were flash-frozen in liquid nitrogen and stored at −80 °C until use.

### *In Vitro* ATPase Assays

ATPase hydrolysis assays were conducted using a Malachite Green Phosphate Assay Kit (Sigma Millipore). Protein samples were diluted to 0.01 mg/mL in a buffer containing 20 mM MOPS/HCl pH 7, 500 mM NaCl, 1 mM DTT, and 0.05% (w/v) DDM. All solutions used in the assay were prepared with Chelex-treated Mili-Q water to minimize trace metals contamination. Each assay sample (200 μL), was obtained by mixing 172 μL of 0.01 mg/mL protein, 16 μL of buffer, 2 μL of a 100X CuCl_2_ stock, 2 μL of TTM (to a final concentration of 50 μM) or DMSO, 4 μL of cysteine (final concentration 2 mM), 2 μL of MgCl_2_ (final concentration 10 mM), and 2 μL of ATP (final concentration 1 mM) to initiate the reaction. The reaction was conducted at 37 °C under continuous agitation for 10 min. Following incubation, 100 μL of the kit’s Working Reagents (Molybdate and Malachite Green) were added to each well to enable color development, proportional to inorganic phosphate (P_*i*_) produced upon ATP hydrolysis. After transferring samples to a 96-well plate, absorbance at 620 nm was measured using a Tecan Spark 20 M plate reader. To quantify P_*i*_, a calibration curve was generated using the P_*i*_ standard provided in the assay kit. Eight standards ranging from 0 to 40 μM were prepared in 200 μL volumes. ATPase activity was calculated and reported as nmol of P_*i*_ per mg of protein per minute.

### *In Vivo* Intracellular Copper Quantification Assays

Overnight cultures of *E. coli* BL21-Gold­(DE3) cells transformed with expression plasmids encoding GS1, GS2, *Ec*CopA, or control (pET-52b­(+)), were inoculated into 35 mL of Terrific Broth (TB) media supplemented with 50 μg/mL ampicillin and grown aerobically until reaching an OD_600_ of 2.0 at 37 °C. Protein expression was induced by adding 0.5 mM IPTG, and the cultures were incubated for 6 h at 30 °C. Subsequently, 10% (v/v) of the cultures was transferred into preweighted tubes containing 5 mL of TB media with ampicillin (50 μg/mL) and CuCl_2_ at final concentrations ranging from 0 mM to 2 mM, with each condition performed in triplicate. These cultures were then grown aerobically under continuous shaking for 10 h overnight at 30 °C. The cells were washed to remove excess extracellular copper by pelleting via centrifugation at 4,000 x*g* for 20 min at 4 °C, resuspending in 5 mL of copper-free TB media, and repeating centrifugation. The resulting cell pellets were dried overnight in a vacuum chamber, and tube weights were recorded to calculate the cell mass. The pellets were then digested in 50% nitric acid (v/v) and incubated at 80 °C overnight. Samples were subsequently diluted to a 3% nitric acid (v/v) before copper quantification via Inductively-Coupled Plasma Mass Spectrometry (ICP-MS) measurements, with intracellular copper levels reported as μg of Cu per mg of cell mass. Metal content was analyzed with an Agilent 7900 ICP mass spectrometer connected to a CETACASX-500 autosampler for sample injection. Statistical significance was evaluated using an unpaired *t*-test, on two independent biological replicates.

### *In Vivo* Copper Susceptibility Growth Assays

To better elucidate the impact of GS1 and GS2 expression in copper extrusion and thereby cellular protection from copper toxicity, copper sensitive *E. coli* strain ECA464 was selected for copper susceptibility toxicity assays.^42^ The copper sensitive strain was subjected to a lysogenization with λDE3 phage following the manufacturer’s user protocol (Novagen cat. 69734). Furthermore, ECA464 λDE3 lysogens were made competent prior to plasmid transformation. The expression of recombinant proteins in this strain was confirmed prior to the assay via Western blot using the previously described protocol (Figure 4A). Thereafter, overnight cultures of transformed cells were inoculated into fresh TB media supplemented with 50 μg/mL ampicillin and grown at 37 °C to an OD_600_ of 2.0. Protein expression was induced by adding 0.5 mM IPTG, and cultures were incubated for an additional 5 h at 30 °C. To monitor the effect of the recombinantly expressed protein, a 96-well plate setup was employed to track OD_600_ cell growth at increasing copper concentrations. Each well was supplemented with TB media containing ampicillin (50 μg/mL), IPTG (0.5 mM) and varying concentrations of CuCl_2_ (0 to 6 mM). Additionally, each well was filled with its corresponding cell culture at a 10% (v/v) dilution. Each construct was tested in triplicate for each copper concentration. OD_600_ was measured in 10-min intervals over 16 h at 37 °C to monitor cell growth using a Tecan Spark 20 M plate reader. See Figure S7B for OD_600_ growth curves. The data was further analyzed by calculating the mean-normalized relative OD_600_ growth for each concentration, using the highest absorbance value recorded at the stationary phase as the reference point (defined as 100% growth). Four biological replicates were performed, and statistical significance was assessed with an unpaired *t*-test.

## Supplementary Material







## Data Availability

The LGL framework used in this study is publicly available at https://github.com/morcoslab/LGL-VAE/. The LGL training data set, and the produced LGL model, a cumulative list of decoded sequences (GS1–GS8) along with MD simulation input files and experimental raw data are uploaded to Zenodo: 10.5281/zenodo.14783470. These files can be accessed through the following link: https://doi.org/10.5281/zenodo.14783470.

## Notes

Additional molecular dynamics analysis, tables, and figures; LGL input MSA summary; sequence alignments with labeled motifs; additional biochemical characterization; MD convergence statistics (PDF)
Simulation movie (MOV)
Simulation movie (MOV)
