# Supplementary material for: Generative Landscapes and Dynamics to Design Functional Multidomain Artificial Transmembrane Transporters
Source: ACS Cent Sci. 2025 Jul 10;11(8):1452–66. doi: 10.1021/acscentsci.5c00708 (PMC12395301; doi:10.1021/acscentsci.5c00708)
Supplement: Supplementary file 1 [file oc5c00708_si_001.pdf]

Supporting Information
Generative Landscapes and Dynamics to Design Functional Multidomain Artificial Transmembrane Transporters

Fernando Montalvillo Ortega[†,‖], Fariha Hossain[‡,‖], Vladimir V. Volobouev[‡], Gabriele Meloni[†,⊥,*], Hedieh Torabifard[†,⊥,*], and Faruck Morcos[‡,¶,§,⊥,*]

[†]Department of Chemistry and Biochemistry, University of Texas at Dallas, Richardson, 75080 TX, USA.

[‡]Department of Biological Sciences, University of Texas at Dallas, Richardson, 75080 TX, USA.

[¶]Departments of Bioengineering and Physics, University of Texas at Dallas, Richardson, 75080 TX, USA.

[§]Center for Systems Biology, University of Texas at Dallas, Richardson, Texas 75080, USA.

[‖]These authors contributed equally to this work.

[⊥]Corresponding author.

[*]Email: gabriele.meloni@utdallas.edu, hedieh.torabifard@utdallas.edu, faruckm@utdallas.edu

List of Supplementary Materials:

SI Text: Molecular dynamics results of additional decoded generated sequences

Figures S1 to S7

Tables S1 to S3

Supplementary Movies S1 to S2

Molecular dynamics results of additional decoded generated sequences

To better understand the impact of the Hamiltonian metric on the generated protein dynamics, we conducted a similar analysis on additional sequences from across the *Lp*CopA area. They were categorized as GS3 to GS8 and their locations on the LGL map and Hamiltonian assigned value can be found in fig. S5, A and D. Regarding their A-domain's ability to undergo conformational changes, several additional decoded sequences did not exhibit large dynamics. First, we observed that various sequences (GS5-GS7) no longer exhibit the characteristic bimodal distribution of the WT in the probability distribution of *A-domain movement scores* presented in fig. S5B. Furthermore, when considering coupled motions between the soluble domain and the TM blocks, half of the additional selected sequences underperform as shown in table S1. Particularly GS4-GS6 do not reach a maximum coupled *A-domain movement score* of 50.00 (half progress). Even when selecting a pliable *A-domain movement score* threshold of 45 based on WT simulation results as well as to accommodate small simulation deviations, the trials of GS4-GS6 were deemed not dynamic enough.

In studying TM helix rearrangement, we found that most of these sequences lacked clear inter-block differentiation (fig. S5D). In several cases, a fraction of the block exhibited significant movement, while the block as a whole did not shift, suggesting a weak intra-block correlation. This observation was quantified by measuring the larger standard deviation between rows and columns per inter-block DDM. Moreover, when contemplating coupled rearrangements between the A-domain and TM domain, approximately half of the trials exhibited underachieving inter-block DDM scores or intra-block correlations. When selecting soft thresholds based on WT simulation results and slight simulation fluctuations, an inter-block DDM score of 1.80 $\AA$ and inter-block DDM standard deviation smaller than 0.75 $\AA$, only GS3 and GS4 displayed consistently significant coupled inter-block DDM scores.

The additional generated sequences (GS3-GS8) combined results of coupled *A-domain movement score* and TM inter-block rearrangement analyses demonstrated subpar inter-domain communication. The same chosen thresholds showcased that half of the WT *Lp*CopA, GS1, and GS2 simulation trials met the requirements whereas the remaining generated sequences did not. These results could indicate that GS3-GS8 decoded sequences could have slow or inefficient coupled dynamics, while GS1 and GS2 larger maximum coupled values resembling WT *Lp*CopA dynamics suggested faster and more coupled dynamics (tables S1 and S2). Thus, to optimize experimental resources, we prioritized GS1 and GS2 for experimental characterization.

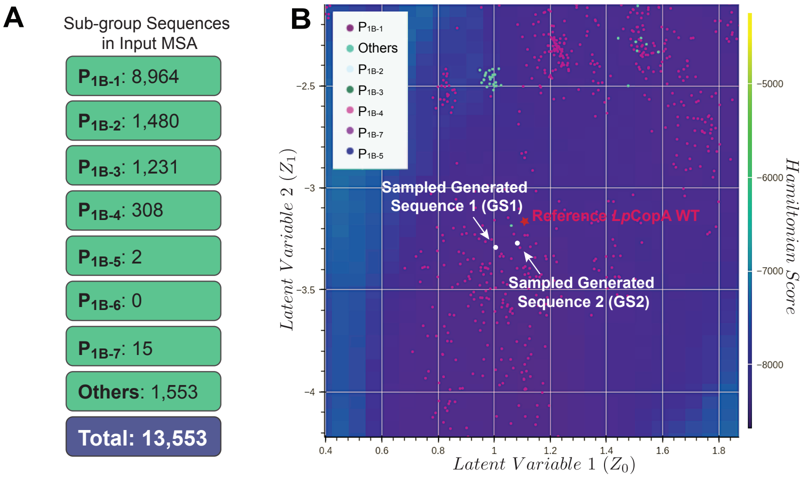

Fig. S1: **LGL training input MSA summary and native sequences placement near GS1 and GS2.** (**A**) Sequence distribution of the P_{1B}-ATPase family used to train the LGL model. (**B**) A 2D close-up of the reference *Lp*CopA WT labeled with a red star. The two selected decoded sequence (GS1 and GS2) locations are labeled with white circles. The nearby colored dots represent other training sequences showing no overlap with the decoded sequences.

```
             MA                                   MB
             1         10        20        30        40        50        60
GS1          YLDMTRRFWIGLALTLPVFVLEMGGHGLHHLIDPQLSNWIQLALATPVVLWAGWPFFVRG
GS2          .VDMTRRFWIGLALTLPVFVLEMGGHGLHHLIGPQLSNWIQLALATPVVLWAGWPFFVRG
LpCopA_WT    YLDMRRRFWIALMLTIPVVILEMGGHGLKHFISGNGSSWIQLLLATPVVLWGGWPFFKRG

                          M1                                M2
                       70        80        90       100       110       120
GS1          WASVRTRNLNMFTLIALGTGVAWLYSVVATLAPGLFPPAFRDHDGAVAVYFEAAAVITVL
GS2          WQSLVTRNLNMFTLIALGTGVAWLYSVVATLAPGLFPPAFRGHDGAVAVYFEAAAVITVL
LpCopA_WT    WQSLKTGQLNMFTLIAMGIGVAWIYSMVAVLWPGVFPHAFRSQEGVVAVYFEAAAVITTL

                          A-Domain
                      130       140       150       160       170       180
GS1          VLLGQVLELRARERTSGAIRALLDLAPKTARRIGADGSEEEVALDQVQVGDRLRVRPGEK
GS2          VLLGQVLELRARERTGGAIRALLDLAPKTARRIGADGSEEEVPLDQVVVGDRLRVRPGEK
LpCopA_WT    VLLGQVLELKAREQTGSAIRALLKLVPESAHRIKEDGSEEEVSLDNVAVGDLLRVRPGEK

                      190       200       210       220       230       240
GS1          VPVDGEVLEGRSSVDESMVTGESMPVTKEVGDKVIGGTINQTGSFVMRAEKVGRDTMLSR
GS2          VPVDGEVLEGRSSVDESMVTGESMPVTKEAGDKVIGGTINQTGSFVMRAEKVGADTMLSQ
LpCopA_WT    IPVDGEVQEGRSFVDESMVTGEPIPVAKEASAKVIGATINQTGSFVMKALHVGSDTMLAR
                                T/SGE

                                M3                              M4
                      250       260       270       280       290       300
GS1          IVQMVAQAQRSRAPIQRLADQVSGWFVPAVIAVALLAFAAWALFGPEPRFSYALIAAVSV
GS2          IVQMVAEAQRSRAPIQRLADQVSGWFVPAVIAVALLAFAAWAIFGPEPAFSYALIAAVSV
LpCopA_WT    IVQMVSDAQRSRAPIQRLADTVSGWFVPAVILVAVLSFIVWALLGPQPALSYGLIAAVSV

                                                 P-Domain
                      310       320       330       340       350       360
GS1          LIIACPCALGLATPMSIMVGVGRGAQAGVLIKNAEALERMEKVDTLVVDKTGTLTEGKPK
GS2          LIIACPCALGLATPMSIMVGVGRGAQAGVLIKNAEALERMEKVDTLVVDKTGTLTEGKPK
LpCopA_WT    LIIACPCALGLATPMSIMVGVGKGAQSGVLIKNAEALERMEKVNTLVVDKTGTLTEGHPK
                 CPC                                      DKTGT

             N-Domain
                      370       380       390       400       410       420
GS1          VTAVVPAAGFDEAELLRLAASLERASEHPLAAAIVAAAEERGLTLAEVEDFDSPTGKGVT
GS2          VTAVVPAAGFAEAELLRLAASLERGSEHPLAAAIVAAAEERGLTLAEVEDFDSPTGKGVT
LpCopA_WT    LTRI.VTDDFVEDNALALAAALEHQSEHPLANAIVHAAKEKGLSLGSVEAFEAPTGKGVV
```

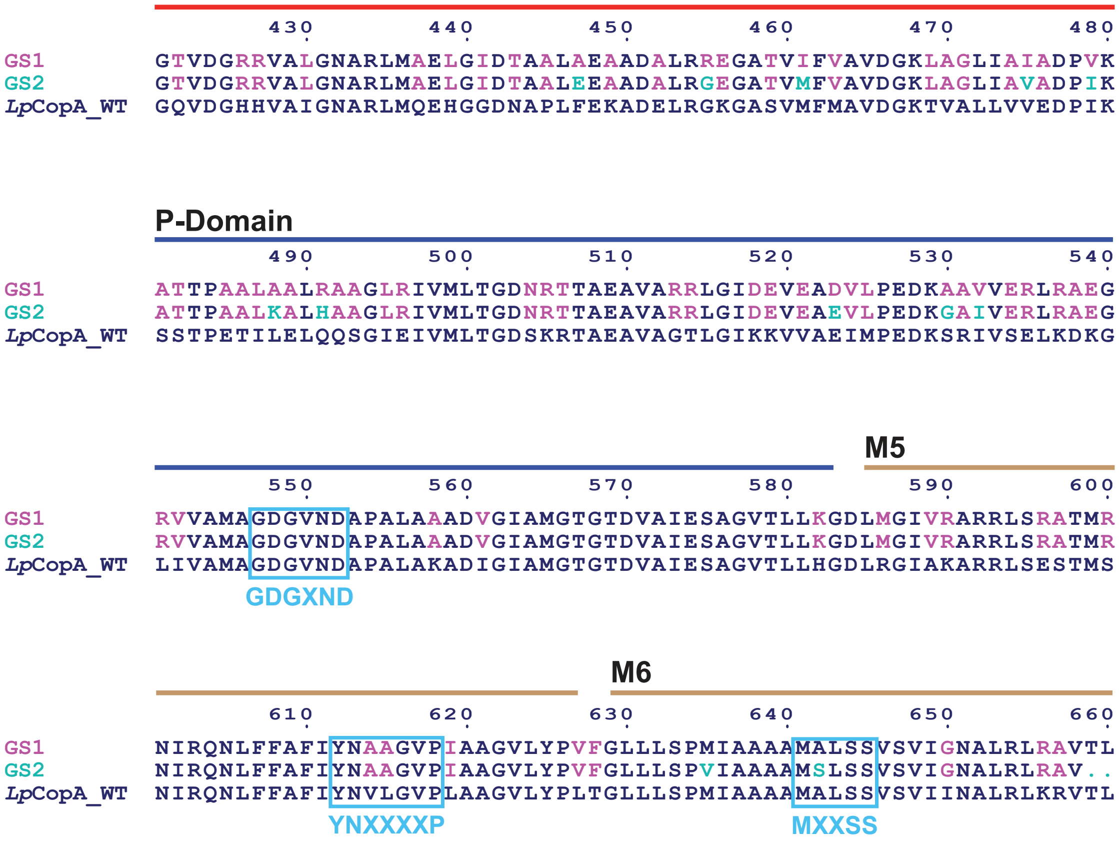

Fig. S2: **Sequence alignment of GS1, GS2, and WT *Lp*CopA**. The color scheme for mutations corresponds to that of Fig. 2C: magenta indicates mutations from *Lp*CopA, while teal highlights the 32 differences between GS1 and GS2. Additionally, domain boundaries and key motifs are labeled for clarity. The LGL decoded sequences were used as alignments against *Lp*CopA without modification, and the figure was generated using ESPript3 [1].

```
                  MA                                          MB
                  1        10        20        30        40        50        60
XtCopA|A0A6I8R0A5 IKQWRNSFLFSLLFGIPVIILMIYMLAANKDHHNTMVLDRNIVPGLSIINLVFFILCTFV
HumanATP7B|P35670 IKQWKKSFLCSLVFGIPVMALMIYMLIPSNEPHQSMVLDHNIIPGLSILNLIFFILCTFV
AfCopA|O29777     ..............................LA.......HFIS..LPYEDFVQLLIALPA
EcCopA|Q59385     ......QAIVALAVGIPVMVWGMI...GDNMM.......V.TA..DNRSLWLVILITLAV
LpCopA_WT|Q5ZWR1  YLDMRRRFWIALMLTIPVVILEMG...GHGLK.......HFIS..GNGSSWIQLLLATPV
GS1               YLDMTRRFWIGLALTLPVFVLEMG...GHGLH.......HLID..PQLSNWIQLALATPV
GS2               .VDMTRRFWIGLALTLPVFVLEMG...GHGLH.......HLIG..PQLSNWIQLALATPV
```

```
                                      M1                                  M2
                           70        80        90       100       110
XtCopA|A0A6I8R0A5 QTLGGRYFYVQAYKSLKHKATNMDVLIVLATTIAYIYSVVILTVAMV.....EKADKSPE
HumanATP7B|P35670 QLLGGWYFYVQAYKSLRHRSANMDVLIVLATSIAYVYSLVILVVAVA.....EKAERSPV
AfCopA|O29777     IFYSGSSIFKAAFSALRRRTLNMDVMYSMGVGAAFLASVLS..TAGVLPREYS.......
EcCopA|Q59385     MVFAGGHFYRSAWKSLLNGAATMDTLVALGTGVAWLYSMSVNLWPQWFPMEAR......H
LpCopA_WT|Q5ZWR1  VLWGGWPFFKRGWQSLKTGQLNMFTLIAMGIGVAWIYSMVAVLWPGVFPHAFRSQEGVVA
GS1               VLWAGWPFFVRGWASVRTRNLNMFTLIALGTGVAWLYSVVATLAPGLFPPAFRDHDGAVA
GS2               VLWAGWPFFVRGWQSLVTRNLNMFTLIALGTGVAWLYSVVATLAPGLFPPAFRGHDGAVA
```

```
                                                A-Domain
                    120       130       140       150       160       170
XtCopA|A0A6I8R0A5 TFFDTPPMLFMFIALGRWLEHIAKSKTSEALAKLISLQATEAAVVTFGANQIILREEQVA
HumanATP7B|P35670 TFFDTPPMLFVFIALGRWLEHLAKSKTSEALAKLMSLQATEATVVTLGEDNLIIREEQVP
AfCopA|O29777     .FYETSVLLLAFLLLGRTLEARAKSRTGEAIKKLVGLQAKTAVVIR.DG.....KEIAVP
EcCopA|Q59385     LYYEASAMIIGLINLGHMLEARARQRSSKALEKLLDLTPPTARLVTDEG.....E.KSVP
LpCopA_WT|Q5ZWR1  VYFEAAAVITTLVLLGQVLELKAREQTGSAIRALLKLVPESAHRIKEDG.....SEEEVS
GS1               VYFEAAAVITVLVLLGQVLELRARERTSGAIRALLDLAPKTARRIGADG.....SEEEVA
GS2               VYFEAAAVITVLVLLGQVLELRARERTGGAIRALLDLAPKTARRIGADG.....SEEEVP
```

```
                    180       190       200       210       220       230
XtCopA|A0A6I8R0A5 VELVQRGDIVKVVPGGKFPVDGKVIEGTSMADESLITGEPMPVRKKPGSMVIAGSINAHG
HumanATP7B|P35670 MELVQRGDIVKVVPGGKFPVDGKVLEGNTMADESLITGEAMPVTKKPGSTVIAGSINAHG
AfCopA|O29777     VEEVAVGDIVIVRPGEKIPVDGVVVEGESYVDESMISGEPVPVLKSKGDEVFGATINNTG
EcCopA|Q59385     LAEVQPGMLLRLTTGDRVPVDGEITQGEAWLDEAMLTGEPIPQQKGEGDSVHAGTVVQDG
LpCopA_WT|Q5ZWR1  LDNVAVGDLLRVRPGEKIPVDGEVQEGRSFVDESMVTGEPIPVAKEASAKVIGATINQTG
GS1               LDQVQVGDRLRVRPGEKVPVDGEVLEGRSSVDESMVTGESMPVTKEVGDKVIGGTINQTG
GS2               LDQVVVGDRLRVRPGEKVPVDGEVLEGRSSVDESMVTGESMPVTKEAGDKVIGGTINQTG
                                                      T/SGE
```

```
                                                         M3
                    240       250       260       270       280       290
XtCopA|A0A6I8R0A5 TVLVEATHVGSETTLAQIVKLVEEAQMSKAPIQQLADKISGYFVPFIIIISVVTLVTWII
HumanATP7B|P35670 SVLIKATHVGNDTTLAQIVKLVEEAQMSKAPIQQLADRFSGYFVPFIIIMSTLTLVVWIV
AfCopA|O29777     VLKIRATRVGGETLLAQIVKLVEDAMGSKPPIQRLADKVVAYFIPTVLLVAISAFIYWYF
EcCopA|Q59385     SVLFRASAVGSHTTLSRIIRMVRQAQSSKPEIGQLADKISAVFVPVVVVIALVSAAIWYF
LpCopA_WT|Q5ZWR1  SFVMKALHVGSDTMLARIVQMVSDAQRSRAPIQRLADTVSGWFVPAVILVAVLSFIVWAL
GS1               SFVMRAEKVGRDTMLSRIVQMVAQAQRSRAPIQRLADQVSGWFVPAVIAVALLAFAAWAL
GS2               SFVMRAEKVGADTMLSQIVQMVAEAQRSRAPIQRLADQVSGWFVPAVIAVALLAFAAWAI
```

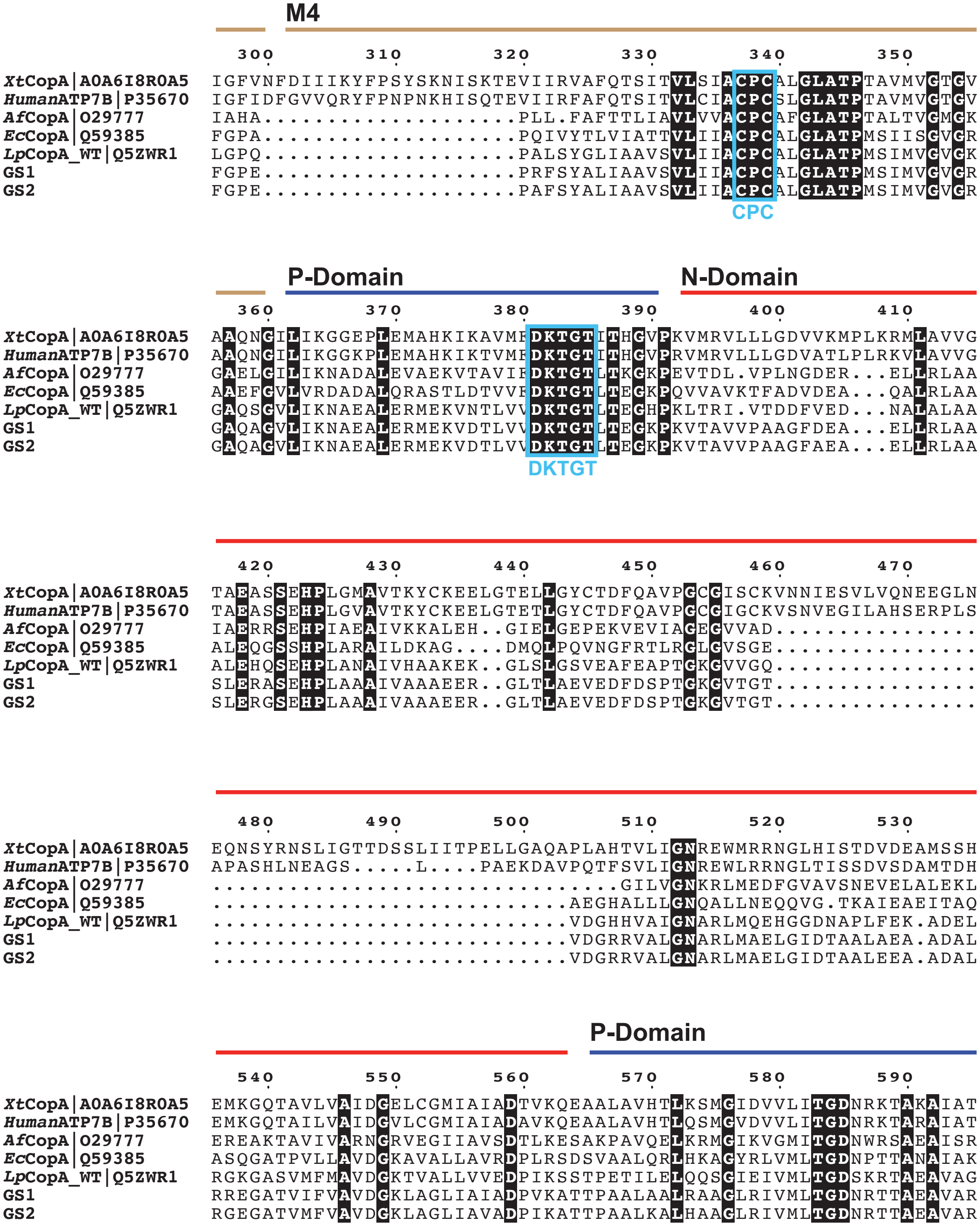
M4
300 310 320 330 340 350
XtCopA|A0A6I8R0A5 IGFVNFDIIIKYFPSYSKNISKTEVIIRVAFQTSITVLSIACPCALGLATPTAVMVGTGV
HumanATP7B|P35670 IGFIDFGVVQRYFPNPNKHISQTEVIIRFAFQTSITVLCIACPCSLGLATPTAVMVGTGV
AfCopA|O29777 IAHA....................PLL.FAFTTLIAVLVVACPCAFGLATPTALTVGMGK
EcCopA|Q59385 FGPA....................PQIVYTLVIATTVLIIACPCALGLATPMSIISGVGR
LpCopA_WT|Q5ZWR1 LGPQ....................PALSYGLIAAVSVLIIACPCALGLATPMSIMVGVGK
GS1 FGPE....................PRFSYALIAAVSVLIIACPCALGLATPMSIMVGVGR
GS2 FGPE....................PAFSYALIAAVSVLIIACPCALGLATPMSIMVGVGR
CPC
P-Domain
N-Domain
360 370 380 390 400 410
XtCopA|A0A6I8R0A5 AAQNGILIKGGEPLEMAHKIKAVMFDKTGTITHGVPKVMRVLLLGDVVKMPLKRMLAVVG
HumanATP7B|P35670 AAQNGILIKGGKPLEMAHKIKTVMFDKTGTITHGVPRVMRVLLLGDVATLPLRKVLAVVG
AfCopA|O29777 GAELGILIKNADALEVAEKVTAVIFDKTGTLTKGKPEVTDL.VPLNGDER...ELLRLAA
EcCopA|Q59385 AAEFGVLVRDADALQRASTLDTVVFDKTGTLTEGKPQVVAVKTFADVDEA...QALRLAA
LpCopA_WT|Q5ZWR1 GAQSGVLIKNAEALERMEKVNTLVVDKTGTLTEGHPKLTRI.VTDDFVED...NALALAA
GS1 GAQAGVLIKNAEALERMEKVDTLVVDKTGTLTEGKPKVTAVVPAAGFDEA...ELLRLAA
GS2 GAQAGVLIKNAEALERMEKVDTLVVDKTGTLTEGKPKVTAVVPAAGFAEA...ELLRLAA
DKTGT
420 430 440 450 460 470
XtCopA|A0A6I8R0A5 TAEASSEHPLGMAVTKYCKEELGTELLGYCTDFQAVPGCGISCKVNNIESVLVQNEEGLN
HumanATP7B|P35670 TAEASSEHPLGVAVTKYCKEELGTETLGYCTDFQAVPGCGIGCKVSNVEGILAHSERPLS
AfCopA|O29777 IAERRSEHPIAEAIVKKALEH..GIELGEPEKVEVIAGEGVVAD................
EcCopA|Q59385 ALEQGSSHPLARAILDKAG....DMQLPQVNGFRTLRGLGVSGE................
LpCopA_WT|Q5ZWR1 ALEHQSEHPLANAIVHAAKEK..GLSLGSVEAFEAPTGKGVVGQ................
GS1 SLERASEHPLAAAIVAAAEER..GLTLAEVEDFDSPTGKGVTGT................
GS2 SLERGSEHPLAAAIVAAAEER..GLTLAEVEDFDSPTGKGVTGT................
480 490 500 510 520 530
XtCopA|A0A6I8R0A5 EQNSYRNSLIGTTDSSLIITPELLGAQAPLAHTVLIGNREWMRRNGLHISTDVDEAMSSH
HumanATP7B|P35670 APASHLNEAGS.....L....PAEKDAVPQTFSVLIGNREWLRRNGLTISSDVSDAMTDH
AfCopA|O29777 ................................GILVGNKRLMEDFGVAVSNEVELALEKL
EcCopA|Q59385 ............................AEGHALLLGNQALLNEQQVG.TKAIEAEITAQ
LpCopA_WT|Q5ZWR1 ............................VDGHHVAIGNARLMQEHGGDNAPLFEK.ADEL
GS1 ............................VDGRRVALGNARLMAELGIDTAALAEA.ADAL
GS2 ............................VDGRRVALGNARLMAELGIDTAALEEA.ADAL
P-Domain
540 550 560 570 580 590
XtCopA|A0A6I8R0A5 EMKGQTAVLVAIDGELCGMIAIADTVKQEAALAVHTLKSMGIDVVLITGDNRKTAKAIAT
HumanATP7B|P35670 EMKGQTAILVAIDGVLCGMIAIADAVKQEAALAVHTLQSMGVDVVLITGDNRKTARAIAT
AfCopA|O29777 EREAKTAVIVARNGRVEGIIAVSDTLKESAKPAVQELKRMGIKVGMITGDNWRSAEAISR
EcCopA|Q59385 ASQGATPVLLAVDGKAVALLAVRDPLRSDSVAALQRLHKAGYRLVMLTGDNPTTANAIAK
LpCopA_WT|Q5ZWR1 RGKGASVMFMAVDGKTVALLVVEDPIKSSTPETILELQQSGIEIVMLTGDSKRTAEAVAG
GS1 RREGATVIFVAVDGKLAGLIAIADPVKATTPAALAALRAAGLRIVMLTGDNRTTAEAVAR
GS2 RGEGATVMFVAVDGKLAGLIAVADPIKATTPAALKALHAAGLRIVMLTGDNRTTAEAVAR

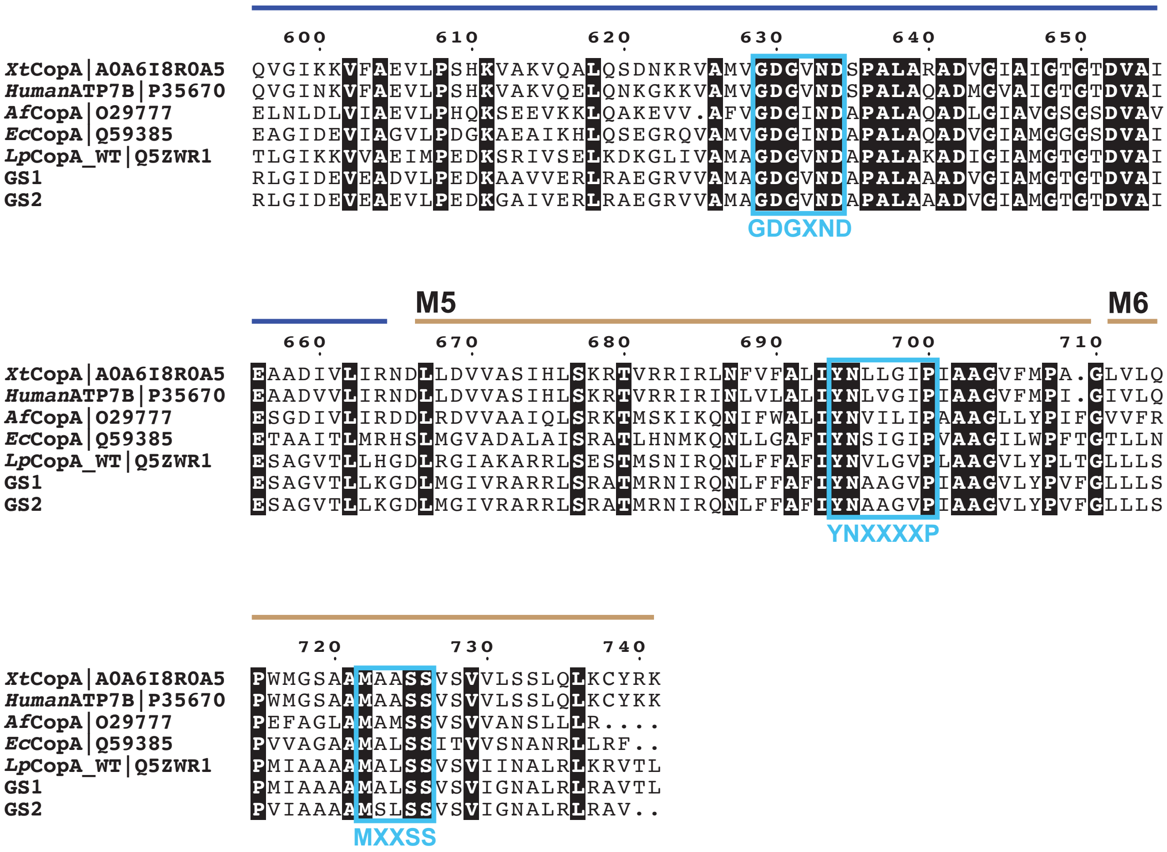

Fig. S3: **Sequence alignment of various characterized P$_{1B-1}$ transporters with GS1 and GS2**. Domain boundaries and key motifs are labeled for clarity and comparison This larger set of sequences was aligned using the Clustal Omega tool hosted by EMBL-EBI, and the final figure was generated using ESPript3 [1–3].

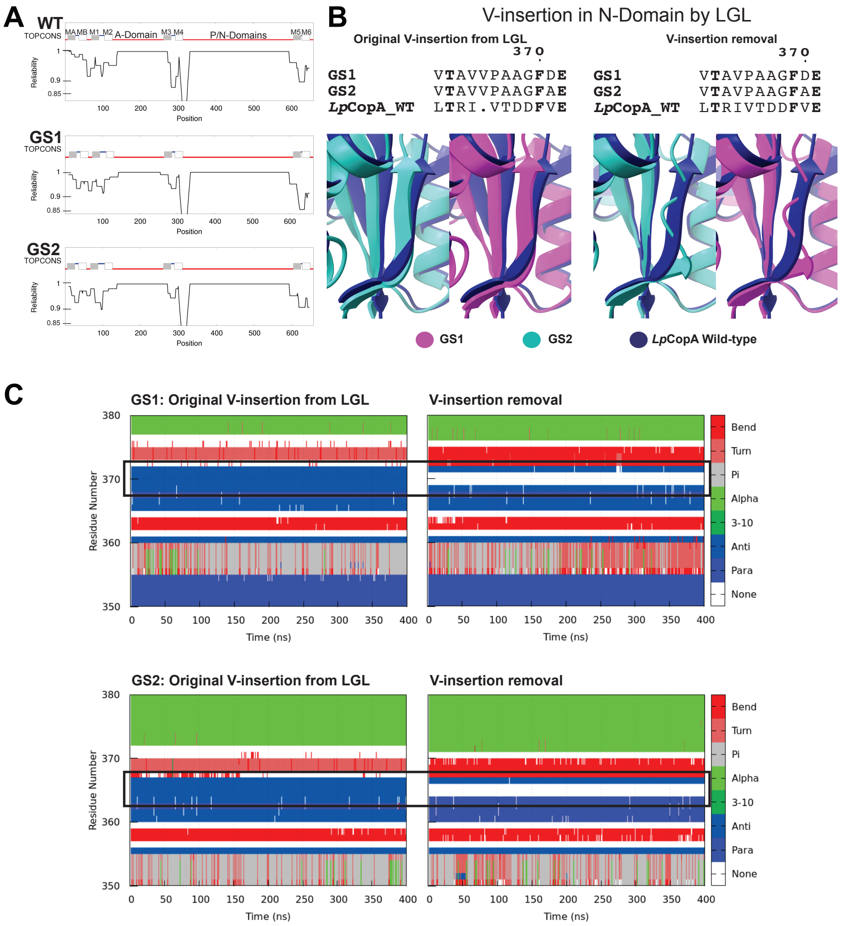

Fig. S4: **Computational pre-screening and closer examination of the V-insertion in the N-domain of GS1 and GS2.** (**A**) TOPCONS, a tool for delineating intracellular and extracellular domains within a sequence [4], predicted a topology for both GS1 and GS2 that closely resembles that of WT *Lp*CopA, featuring eight transmembrane helices and two extensive intracellular regions. (**B**) AF2-predicted structures demonstrate that the V-insertion (valine) in both GS1 and GS2 is essential for the proper formation of the first N-domain β-sheet (P359 to A367 in GS1 and P358 to A366 in GS2) [5]. (**C**) Secondary structure analysis indicates a failure to retrieve the corresponding β-sheet during simulations, as evidenced by the absence of secondary structure in the black box bounded region.

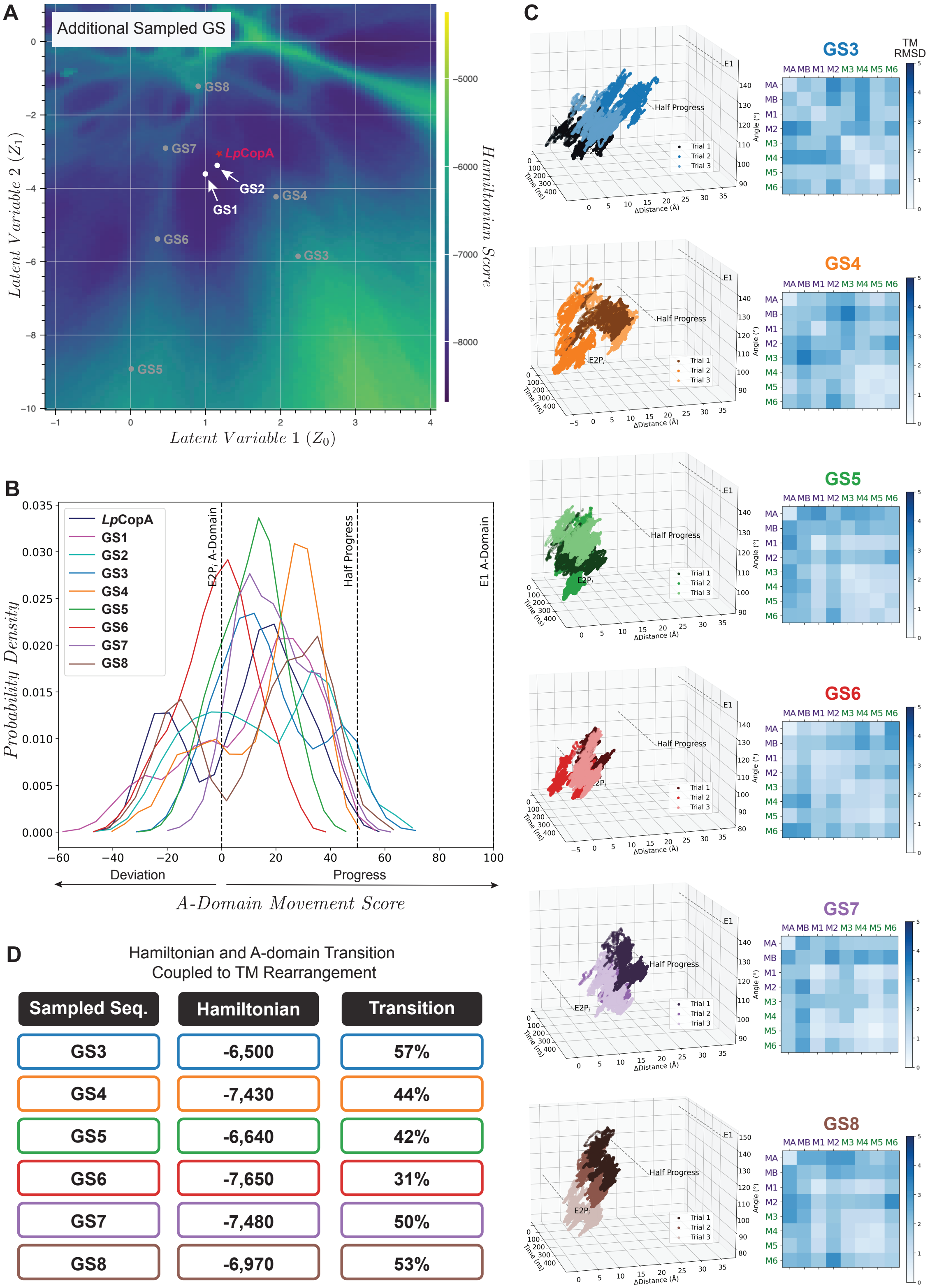

Fig. S5: **MD analysis of additional generated sequences** (**A**) Decoding location of GS3-GS8. (**B**) Comparison of *A-domain movement score* reveals that most generated sequences fail to replicate the results of WT. (**C**) TM rearrangements were relatively weak, with two-block movements notably absent. (**D**) Hamiltonian values from LGL and the coupled A-domain transition progress corresponding to the reported TM rearrangement that is shown in part C.

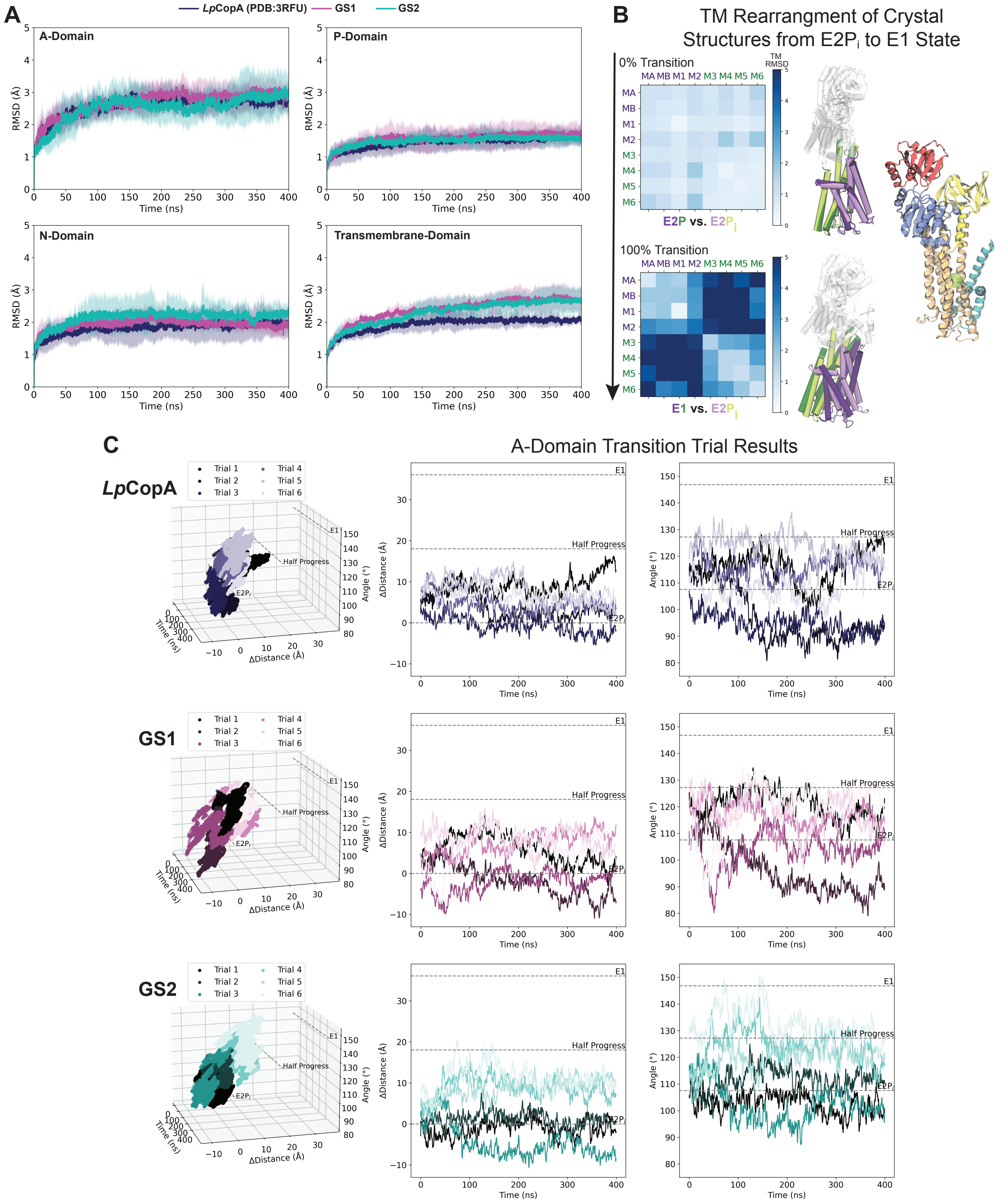

Fig. S6: **Additional MD simulation details for *Lp*CopA, GS1 and GS2.** (**A**) Average RMSD comparisons of the different domains derived from MD simulations to monitor system stability, calculated with respect to the initial frame of each simulation. (**B**) Complete transmembrane rearrangement coupled to the A-domain transition, as determined from crystal structures of the E2P_i (PDB: 3RFU; *Legionella pneumophila*, *Lp*CopA) and early-E1 (PDB: 7R0I; *Archaeoglobus fulgidus*, *Af*CopA) states. (**C**) Results from all six simulation trials that were utilized to determine the *A-domain movement score* from the E2P_i to the E1 state in Fig. 3D.

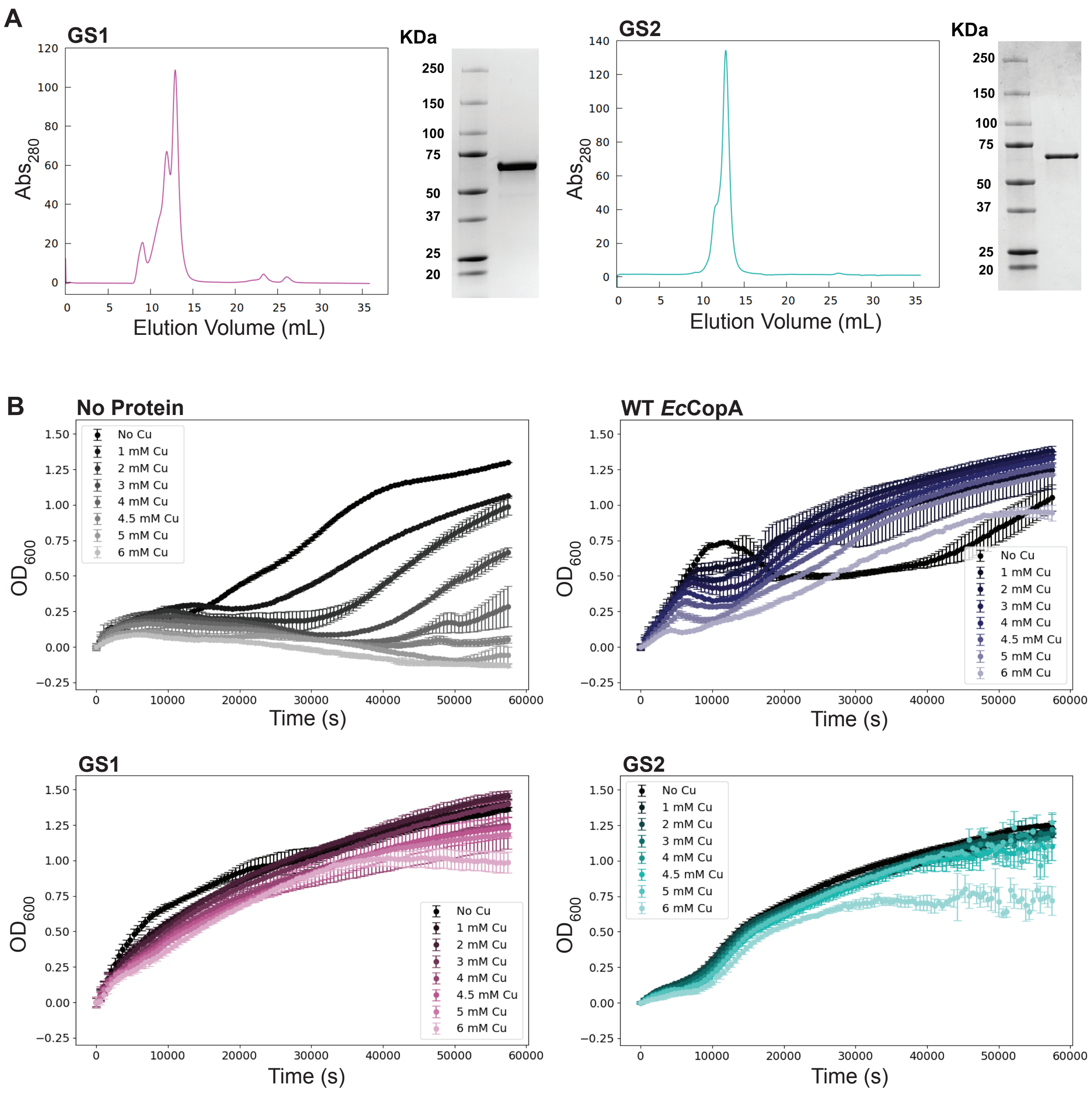

Fig. S7: **Generated sequences purification and additional biochemical characterization.** (**A**) Size exclusion chromatography analysis of purified GS1 and GS2 with corresponding SDS-PAGE. (**B**) OD_{600} results from a representative copper susceptibility growth assay

Table S1. Maximum coupled *A-domain movement score* achieved across trials.

	Structure	Trial 1	Trial 2	Trial 3	Trial 4	Trial 5	Trial 6
A-domain movement score - Progress (%)	E2P (CS)	0.00	–	–	–	–	–
	E1 (CS)	100.00	–	–	–	–	–
	WT	52.91	30.99	3.64	47.26	57.78	36.47
	GS1	50.64	20.60	15.51	45.39	51.37	51.05
	GS2	11.25	19.96	25.84	53.74	46.16	64.49
	GS3	22.72	57.11	25.03	–	–	–
	GS4	43.35	32.14	43.54	–	–	–
	GS5	35.56	41.58	34.23	–	–	–
	GS6	18.62	8.12	31.19	–	–	–
	GS7	50.03	28.48	28.45	–	–	–
	GS8	53.02	49.85	12.39	–	–	–

Table S2. Maximum coupled inter-block helix DDM achieved across trials. Parenthetical values show the larger standard deviation between rows and columns in the inter-block DDM.

	Structure	Trial 1	Trial 2	Trial 3	Trial 4	Trial 5	Trial 6
Averaged Inter-block RMSD (Å)	E2P (CS)	1.12 (0.38)	–	–	–	–	–
	E1 (CS)	5.07 (0.51)	–	–	–	–	–
	WT	1.93 (0.43)	1.47 (0.45)	1.57 (0.24)	1.83 (0.38)	1.82 (0.25)	1.53 (0.49)
	GS1	1.35 (0.48)	1.25 (0.24)	1.13 (0.22)	1.75 (0.41)	2.19 (0.31)	1.86 (0.62)
	GS2	1.02 (0.17)	1.13 (0.33)	1.33 (0.58)	2.56 (0.75)	2.75 (0.38)	2.26 (0.66)
	GS3	1.99 (0.32)	2.23 (0.46)	2.13 (0.46)	–	–	–
	GS4	2.07 (0.72)	2.37 (0.53)	2.17 (0.43)	–	–	–
	GS5	1.61 (0.25)	2.06 (0.18)	1.75 (0.23)	–	–	–
	GS6	1.67 (0.41)	1.42 (0.25)	2.06 (0.37)	–	–	–
	GS7	1.78 (0.57)	2.57 (1.02)	2.03 (0.41)	–	–	–
	GS8	2.06 (0.29)	1.72 (0.45)	1.78 (0.39)	–	–	–

Table S3. Unit root test results perform on RMSD per domain traces to confirm converge and stationary state (p-values below 0.05 are stationary)

	**System**	**Domain**	**Trial 1**	**Trial 2**	**Trial 3**	**Trial 4**	**Trial 5**	**Trial 6**
Unit root test p-values	WT	A	0.0014	0.0033	0.0001	0.0000	0.0000	0.0000
	GS1	A	0.0000	0.0009	0.0000	0.0000	0.0000	0.0000
	GS2	A	0.0000	0.0000	0.0040	0.0000	0.0003	0.0004
	WT	P	0.0024	0.0362	0.0002	0.0001	0.0005	0.0000
	GS1	P	0.0000	0.0000	0.0002	0.0000	0.0000	0.0002
	GS2	P	0.0000	0.0000	0.0000	0.0000	0.0000	0.0000
	WT	N	0.0000	0.0000	0.0000	0.0014	0.0000	0.0035
	GS1	N	0.0000	0.0000	0.0000	0.0012	0.0000	0.0000
	GS2	N	0.0000	0.0004	0.0000	0.0007	0.0000	0.0000
	WT	TM	0.0101	0.0000	0.0000	0.0000	0.0000	0.0000
	GS1	TM	0.0380	0.0002	0.0258	0.0007	0.0000	0.0000
	GS2	TM	0.0165	0.0130	0.0002	0.0003	0.0006	0.0000

Supplementary Movie S1. Representative simulation trial showcasing A-domain dynamics over 400 ns simulation by tracking changes in tilt angle and Δdistance for GS2.

Supplementary Movie S2. Representative simulation trial showcasing transmembrane helices rearrangement by tracking the Distance Difference Matrix over 400 ns simulation for GS2.

References

[1] Xavier Robert and Patrice Gouet. Deciphering key features in protein structures with the new endscript server. *Nucleic Acids Research*, 42(W1):W320–W324, 2014.

[2] Fabian Sievers, Andreas Wilm, David Dineen, Toby J Gibson, Kevin Karplus, Weizhong Li, Rodrigo Lopez, Hamish McWilliam, Michael Remmert, Johannes Söding, et al. Fast, scalable generation of high-quality protein multiple sequence alignments using clustal omega. *Molecular Systems Biology*, 7(1):539, 2011.

[3] Mickael Goujon, Hamish McWilliam, Weizhong Li, Franck Valentin, Silvano Squizzato, Juri Paern, and Rodrigo Lopez. A new bioinformatics analysis tools framework at embl–ebi. *Nucleic Acids Research*, 38(suppl_2):W695–W699, 2010.

[4] KD Tsirigos, C Peters, N Shu, L Käll, and A Elofsson. The topcons web server for combined membrane protein topology and signal peptide prediction. *Nucleic Acids Research*, 43:W401–W407, 2015.

[5] John Jumper, Richard Evans, Alexander Pritzel, Tim Green, Michael Figurnov, Olaf Ronneberger, Kathryn Tunyasuvunakool, Russ Bates, Augustin Žídek, Anna Potapenko, et al. Highly accurate protein structure prediction with alphafold. *Nature*, 596(7873):583–589, 2021.
